# Supplementary material for: Isothermal titration calorimetry and surface plasmon resonance analysis using the dynamic approach
Source: Biochem Biophys Rep. 2019 Dec 17;21:100712. doi: 10.1016/j.bbrep.2019.100712 (PMC6926116; doi:10.1016/j.bbrep.2019.100712)
Supplement: Multimedia component 2 [file mmc2.zip › Figure_2/MNOS_Five_mixed/Time_domain/Chromatogram.pdf]

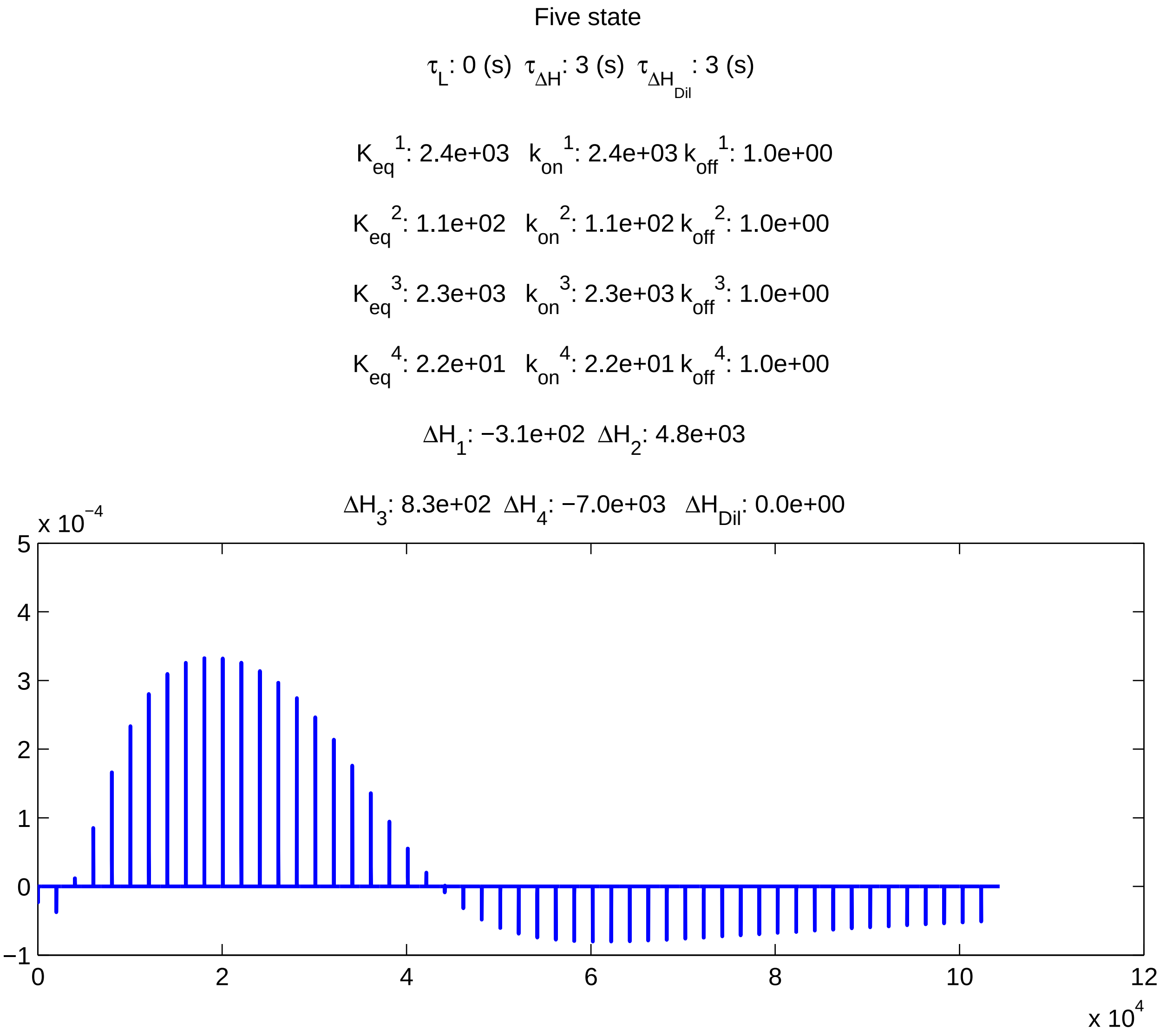
Five state
τL: 0 (s) τΔH: 3 (s) τΔHDil: 3 (s)
Keq1: 2.4e+03 kon1: 2.4e+03 koff1: 1.0e+00
Keq2: 1.1e+02 kon2: 1.1e+02 koff2: 1.0e+00
Keq3: 2.3e+03 kon3: 2.3e+03 koff3: 1.0e+00
Keq4: 2.2e+01 kon4: 2.2e+01 koff4: 1.0e+00
ΔH1: −3.1e+02 ΔH2: 4.8e+03
ΔH3: 8.3e+02 ΔH4: −7.0e+03 ΔHDil: 0.0e+00
x 10−4
5
4
3
2
1
0
−1
0
2
4
6
8
10
12
x 104
