# Supplementary material for: Isothermal titration calorimetry and surface plasmon resonance analysis using the dynamic approach
Source: Biochem Biophys Rep. 2019 Dec 17;21:100712. doi: 10.1016/j.bbrep.2019.100712 (PMC6926116; doi:10.1016/j.bbrep.2019.100712)
Supplement: Multimedia component 2 [file mmc2.zip › Figure_2/MNOS_Five_mixed/Time_domain/Processed_data.pdf]

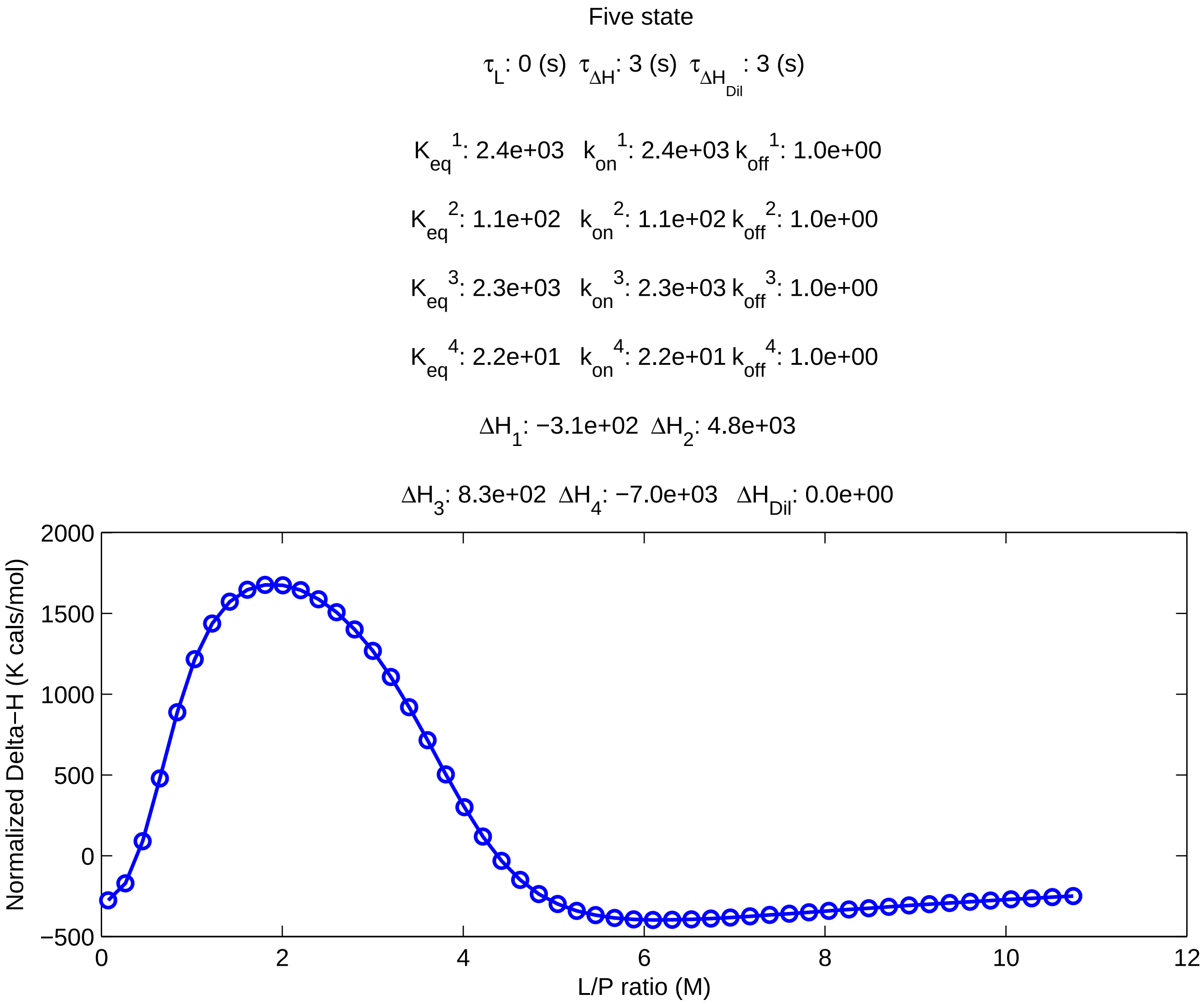

Five state
τ_L: 0 (s) $\tau_{\Delta H}$: 3 (s) $\tau_{\Delta H_{Dil}}$: 3 (s)
K_{eq}^1: 2.4e+03 k_{on}^1: 2.4e+03 k_{off}^1: 1.0e+00
K_{eq}^2: 1.1e+02 k_{on}^2: 1.1e+02 k_{off}^2: 1.0e+00
K_{eq}^3: 2.3e+03 k_{on}^3: 2.3e+03 k_{off}^3: 1.0e+00
K_{eq}^4: 2.2e+01 k_{on}^4: 2.2e+01 k_{off}^4: 1.0e+00
ΔH_1: −3.1e+02 ΔH_2: 4.8e+03
ΔH_3: 8.3e+02 ΔH_4: −7.0e+03 ΔH_{Dil}: 0.0e+00
Normalized Delta−H (K cals/mol)
2000
1500
1000
500
0
−500
0
2
4
6
8
10
12
L/P ratio (M)
