# Supplementary material for: Isothermal titration calorimetry and surface plasmon resonance analysis using the dynamic approach
Source: Biochem Biophys Rep. 2019 Dec 17;21:100712. doi: 10.1016/j.bbrep.2019.100712 (PMC6926116; doi:10.1016/j.bbrep.2019.100712)
Supplement: Multimedia component 2 [file mmc2.zip › Figure_2/MN_Independent/Time_domain/Chromatogram.pdf]

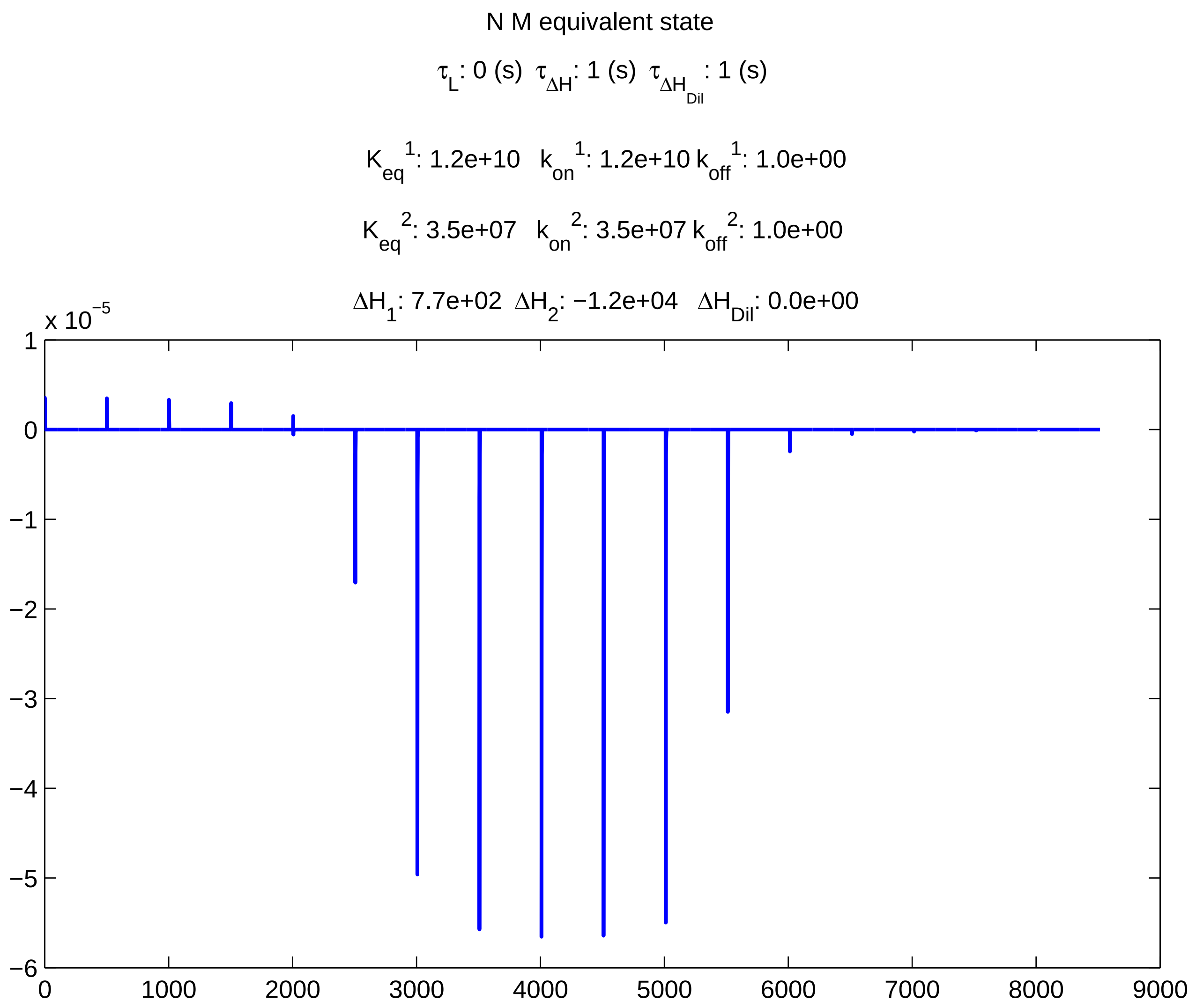

N M equivalent state
τ_L: 0 (s) $\tau_{\Delta H}$: 1 (s) $\tau_{\Delta H_{Dil}}$: 1 (s)
K_{eq}^1: 1.2e+10 k_{on}^1: 1.2e+10 k_{off}^1: 1.0e+00
K_{eq}^2: 3.5e+07 k_{on}^2: 3.5e+07 k_{off}^2: 1.0e+00
ΔH_1: 7.7e+02 ΔH_2: −1.2e+04 ΔH_{Dil}: 0.0e+00
x 10^{-5}
1
0
−1
−2
−3
−4
−5
−6
0
1000
2000
3000
4000
5000
6000
7000
8000
9000
