# Supplementary material for: Isothermal titration calorimetry and surface plasmon resonance analysis using the dynamic approach
Source: Biochem Biophys Rep. 2019 Dec 17;21:100712. doi: 10.1016/j.bbrep.2019.100712 (PMC6926116; doi:10.1016/j.bbrep.2019.100712)
Supplement: Multimedia component 2 [file mmc2.zip › Figure_2/MN_Independent/Time_domain/Processed_data.pdf]

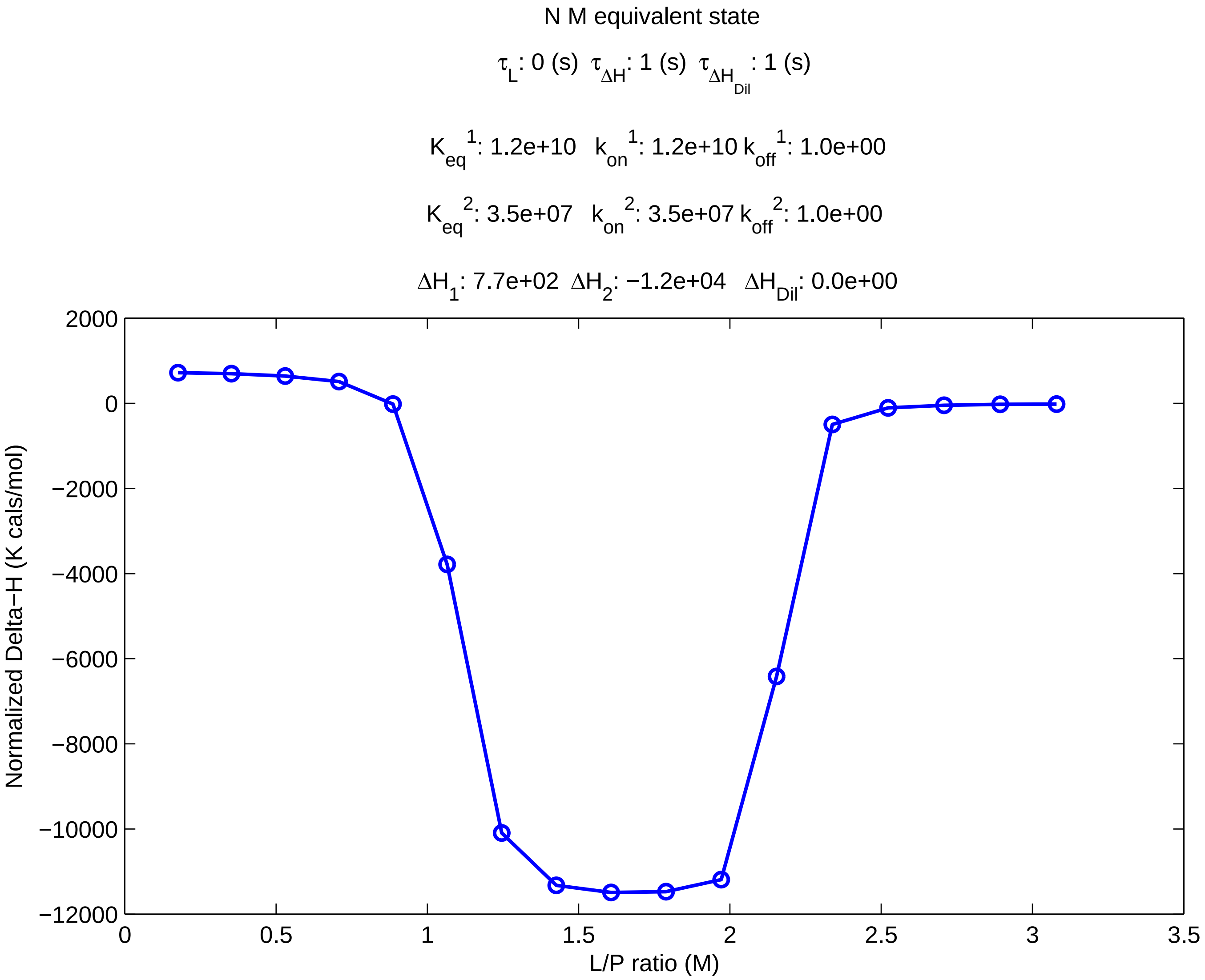
N M equivalent state
$\tau_L$: 0 (s) $\tau_{\Delta H}$: 1 (s) $\tau_{\Delta H_{Dil}}$: 1 (s)
$K_{eq}^1$: 1.2e+10 $k_{on}^1$: 1.2e+10 $k_{off}^1$: 1.0e+00
$K_{eq}^2$: 3.5e+07 $k_{on}^2$: 3.5e+07 $k_{off}^2$: 1.0e+00
$\Delta H_1$: 7.7e+02 $\Delta H_2$: −1.2e+04 $\Delta H_{Dil}$: 0.0e+00
Normalized Delta−H (K cals/mol)
2000
0
−2000
−4000
−6000
−8000
−10000
−12000
0
0.5
1
1.5
2
2.5
3
3.5
L/P ratio (M)
