# Supplementary material for: Isothermal titration calorimetry and surface plasmon resonance analysis using the dynamic approach
Source: Biochem Biophys Rep. 2019 Dec 17;21:100712. doi: 10.1016/j.bbrep.2019.100712 (PMC6926116; doi:10.1016/j.bbrep.2019.100712)
Supplement: Multimedia component 2 [file mmc2.zip › Figure_2/MN_Three_mixed/Time_domain/With_IR/Processed_data.pdf]

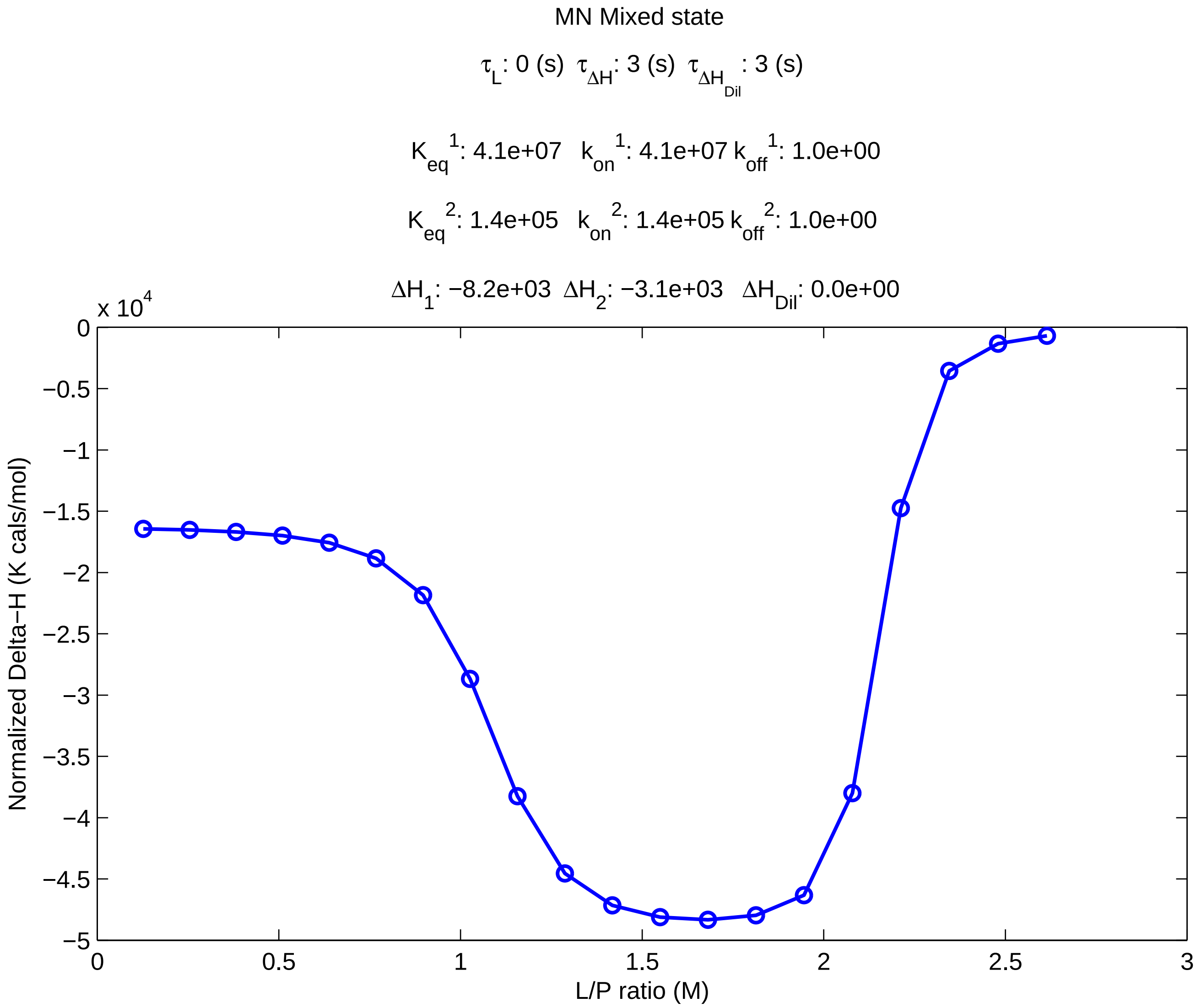
MN Mixed state
τL: 0 (s) τΔH: 3 (s) τΔHDil: 3 (s)
Keq1: 4.1e+07 kon1: 4.1e+07 koff1: 1.0e+00
Keq2: 1.4e+05 kon2: 1.4e+05 koff2: 1.0e+00
ΔH1: −8.2e+03 ΔH2: −3.1e+03 ΔHDil: 0.0e+00
x 10^4
0
−0.5
−1
−1.5
−2
−2.5
−3
−3.5
−4
−4.5
−5
Normalized Delta−H (K cals/mol)
0
0.5
1
1.5
2
2.5
3
L/P ratio (M)
