# Supplementary material for: Isothermal titration calorimetry and surface plasmon resonance analysis using the dynamic approach
Source: Biochem Biophys Rep. 2019 Dec 17;21:100712. doi: 10.1016/j.bbrep.2019.100712 (PMC6926116; doi:10.1016/j.bbrep.2019.100712)

MN Mixed Three state

τ_L: 0 (s) $\tau_{\Delta H}$: 3 (s) $\tau_{\Delta H_{Dil}}$: 3 (s)

K_{eq}^1: 4.1e+07 k_{on}^1: 4.1e+07 k_{off}^1: 1.0e+00

K_{eq}^2: 1.4e+05 k_{on}^2: 1.4e+05 k_{off}^2: 1.0e+00

ΔH_1: −8.2e+03 ΔH_2: −3.1e+03 ΔH_{Dil}: 0.0e+00

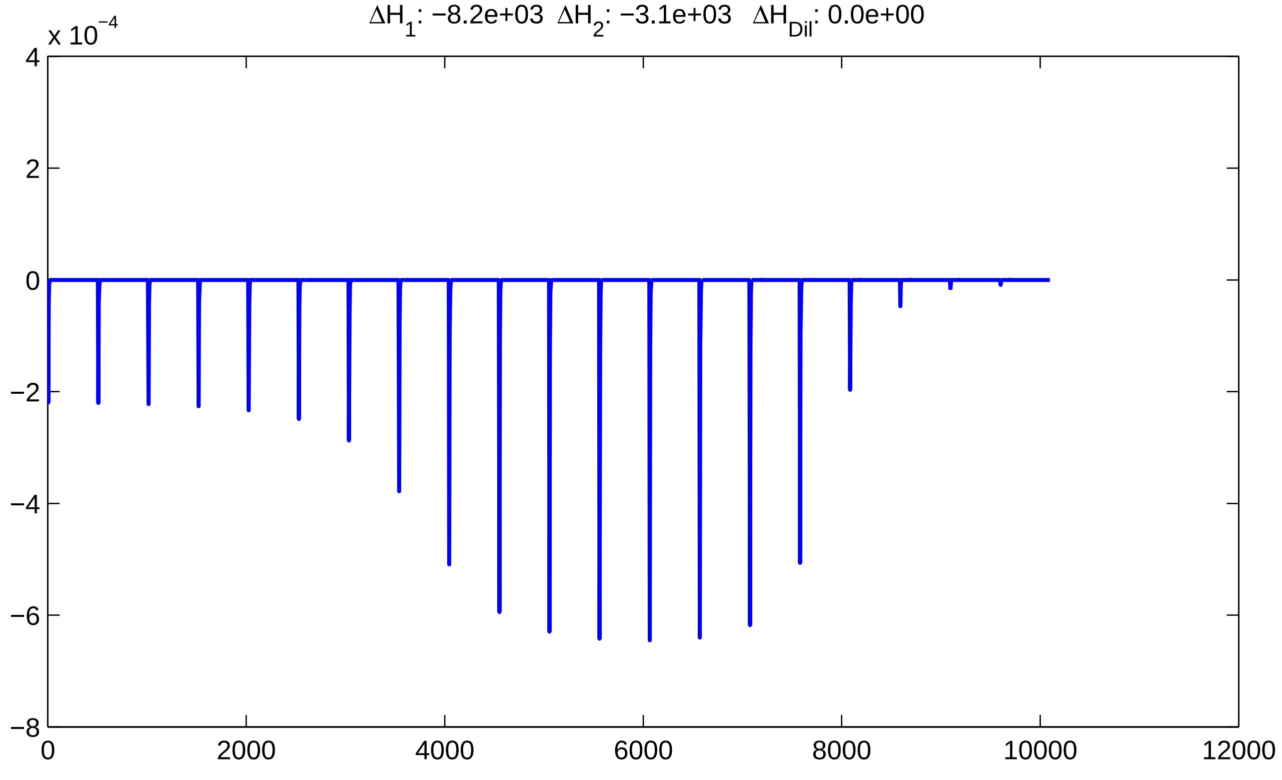

Supplement: Multimedia component 2 [file mmc2.zip › Figure_2/MN_Three_mixed/Time_domain/With_IR_1/Chromatogram.pdf]
