# Supplementary material for: Isothermal titration calorimetry and surface plasmon resonance analysis using the dynamic approach
Source: Biochem Biophys Rep. 2019 Dec 17;21:100712. doi: 10.1016/j.bbrep.2019.100712 (PMC6926116; doi:10.1016/j.bbrep.2019.100712)
Supplement: Multimedia component 2 [file mmc2.zip › Figure_2/MN_Three_mixed/Time_domain/Without_IR/Chromatogram.pdf]

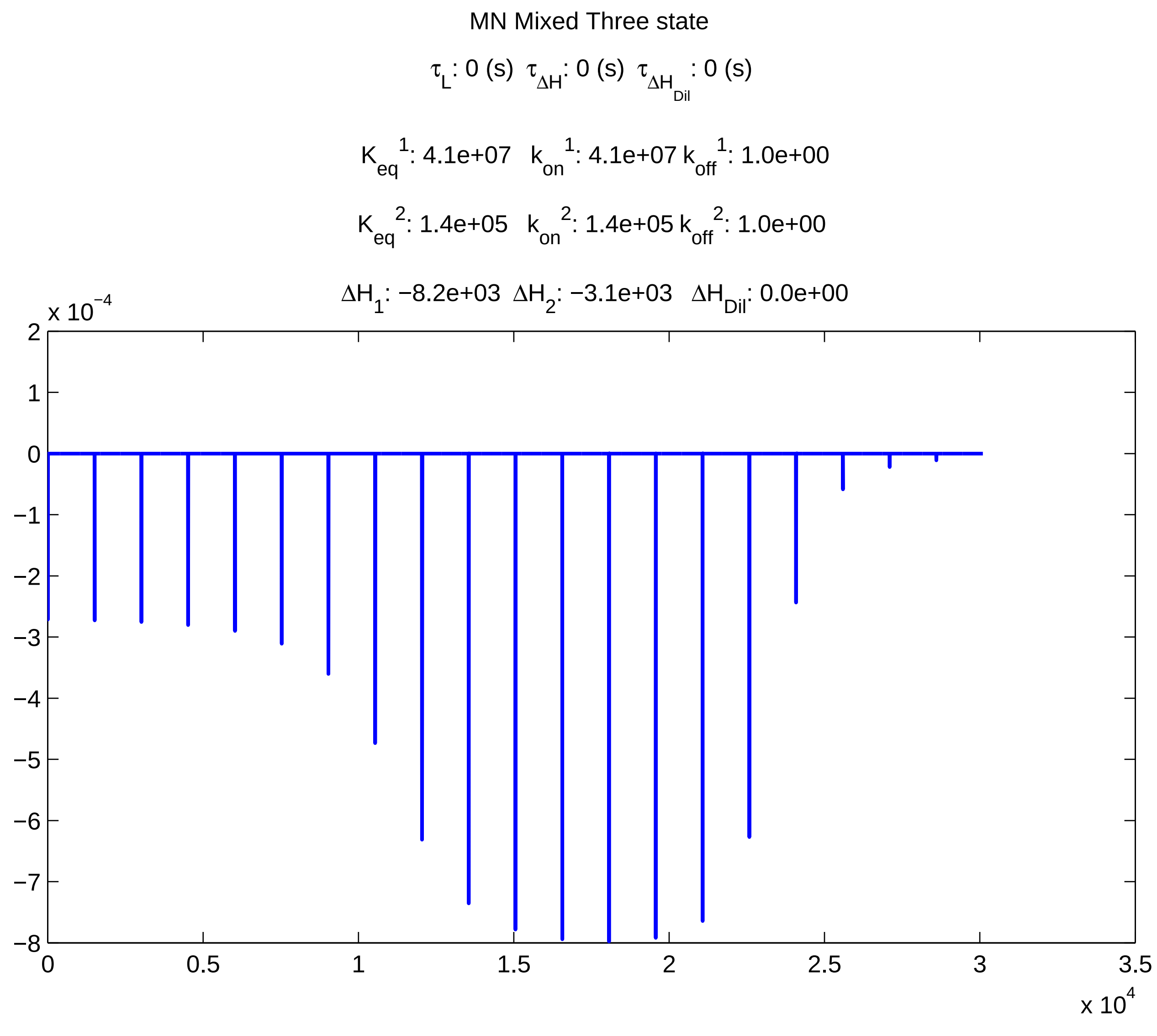
MN Mixed Three state
τL: 0 (s) τΔH: 0 (s) τΔHDil: 0 (s)
Keq1: 4.1e+07 kon1: 4.1e+07 koff1: 1.0e+00
Keq2: 1.4e+05 kon2: 1.4e+05 koff2: 1.0e+00
ΔH1: −8.2e+03 ΔH2: −3.1e+03 ΔHDil: 0.0e+00
x 10−4
2
1
0
−1
−2
−3
−4
−5
−6
−7
−8
0
0.5
1
1.5
2
2.5
3
3.5
x 104
