# Supplementary figures and images for: Isothermal titration calorimetry and surface plasmon resonance analysis using the dynamic approach
Source: Biochem Biophys Rep. 2019 Dec 17;21:100712. doi: 10.1016/j.bbrep.2019.100712 (PMC6926116; doi:10.1016/j.bbrep.2019.100712)

Two state

$\tau_L: 3 \text{ (s)}$   $\tau_{\Delta H}: 3 \text{ (s)}$   $\tau_{\Delta H_{Dil}}: 3 \text{ (s)}$

$K_{eq}: 1.0e+06$   $k_{on}: 1.0e+06$   $k_{off}: 1.0e+00$

$\Delta H: -1.0e+04$   $\Delta H_{Dil}: -1.0e+02$

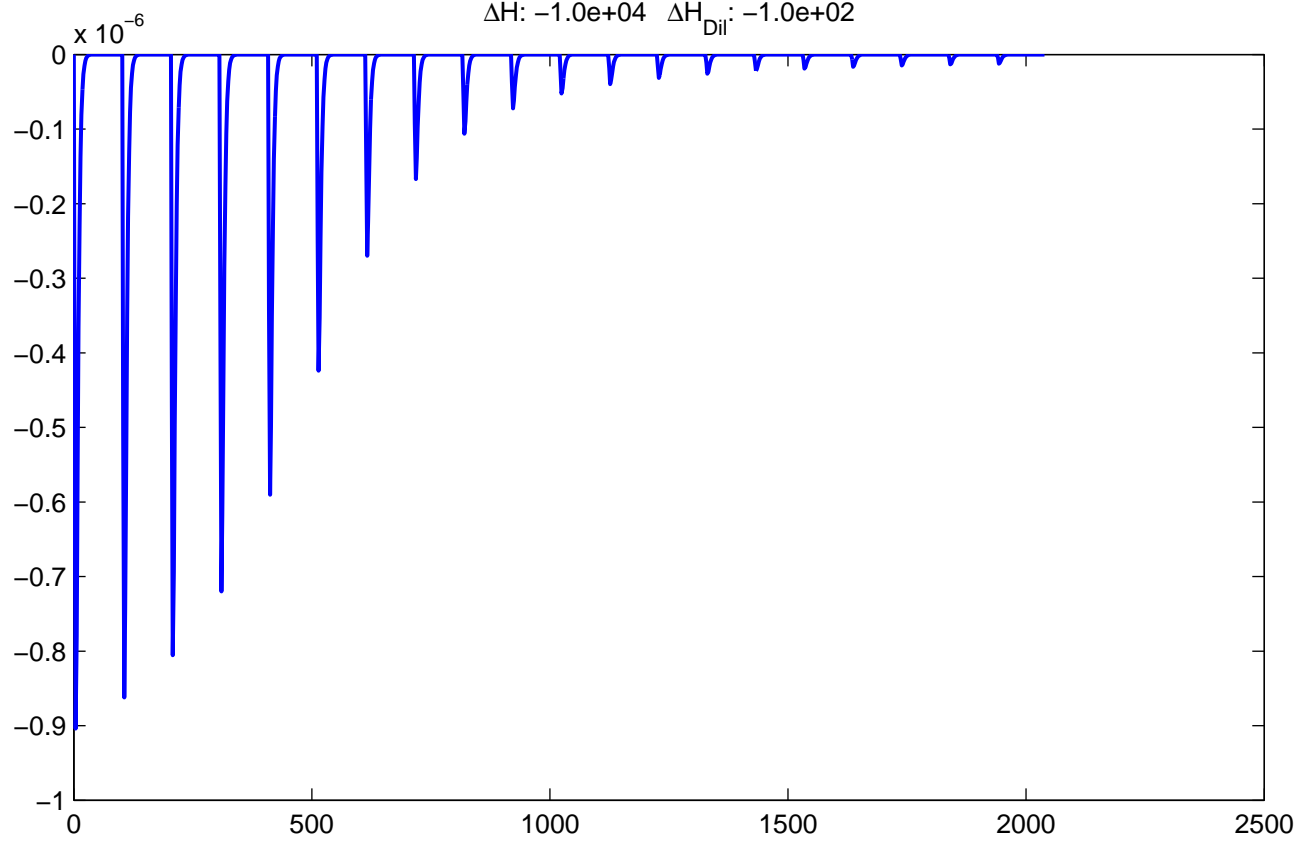

Supplement: Multimedia component 2 [file mmc2.zip › Figure_1/Kinetic_Parallel/Chromatogram.pdf]

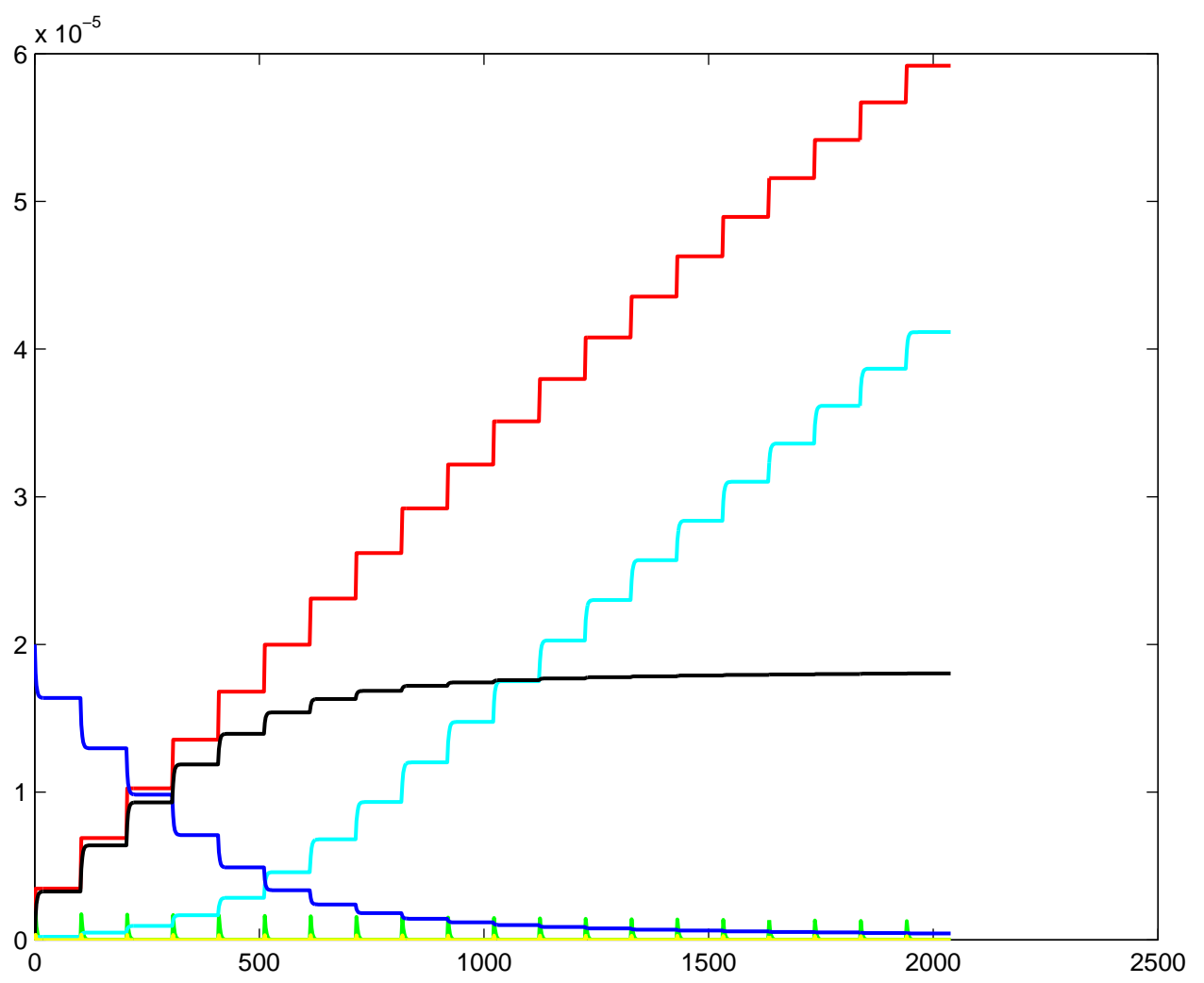

Supplement: Multimedia component 2 [file mmc2.zip › Figure_1/Kinetic_Parallel/Kinetics(P,PL,L).pdf]

Two state

$\tau_L$ : 3 (s)  $\tau_{\Delta H}$ : 3 (s)  $\tau_{\Delta H_{Dil}}$ : 3 (s)

$K_{eq}$ : 1.0e+06  $k_{on}$ : 1.0e+06  $k_{off}$ : 1.0e+00

$\Delta H$ : -1.0e+04  $\Delta H_{Dil}$ : -1.0e+02

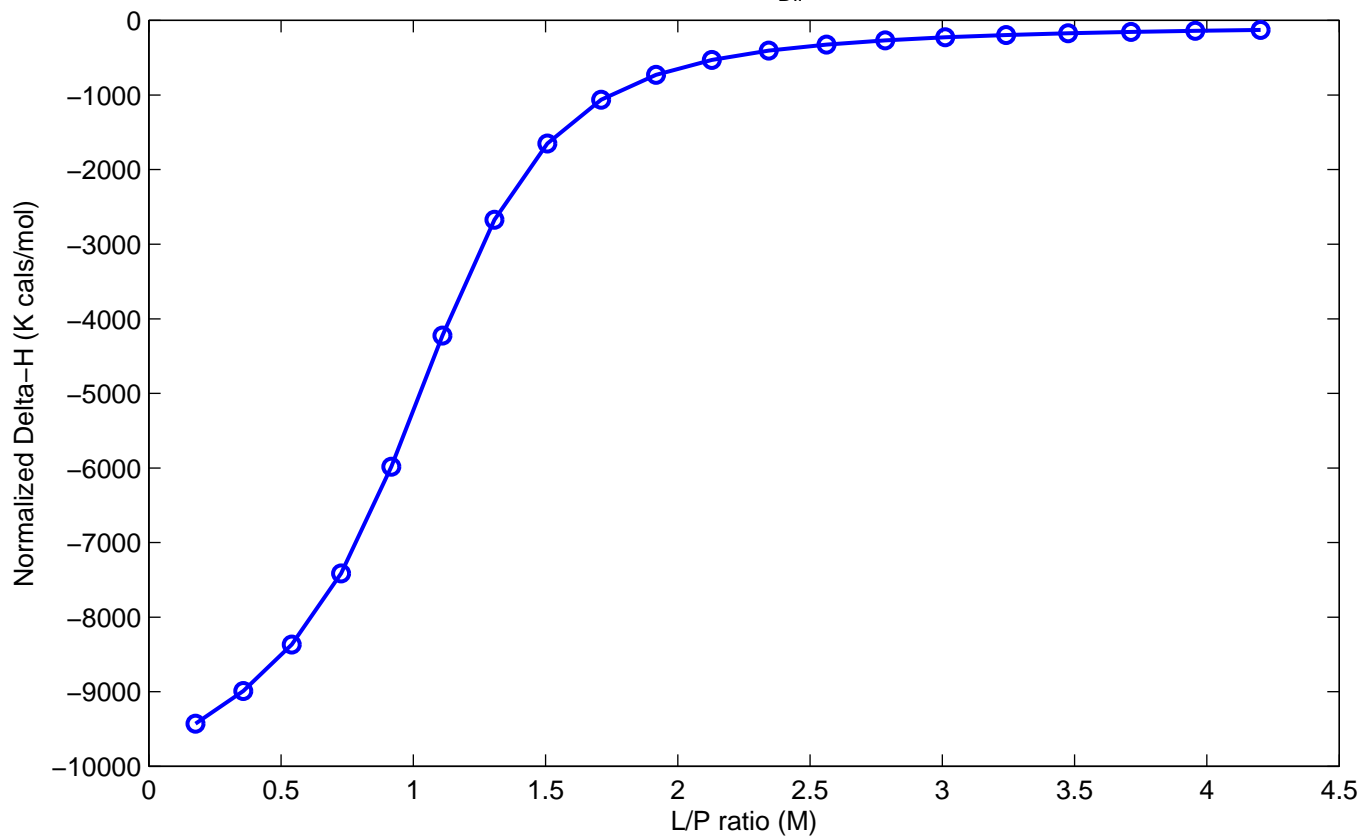

Supplement: Multimedia component 2 [file mmc2.zip › Figure_1/Kinetic_Parallel/Processed_data.pdf]

Two state

$\tau_L: 3 \text{ (s)}$   $\tau_{\Delta H}: 3 \text{ (s)}$   $\tau_{\Delta H_{Dil}}: 3 \text{ (s)}$

$K_{eq}: 1.0e+06$   $k_{on}: 1.0e+06$   $k_{off}: 1.0e+00$

$\Delta H: -1.0e+04$   $\Delta H_{Dil}: -1.0e+02$

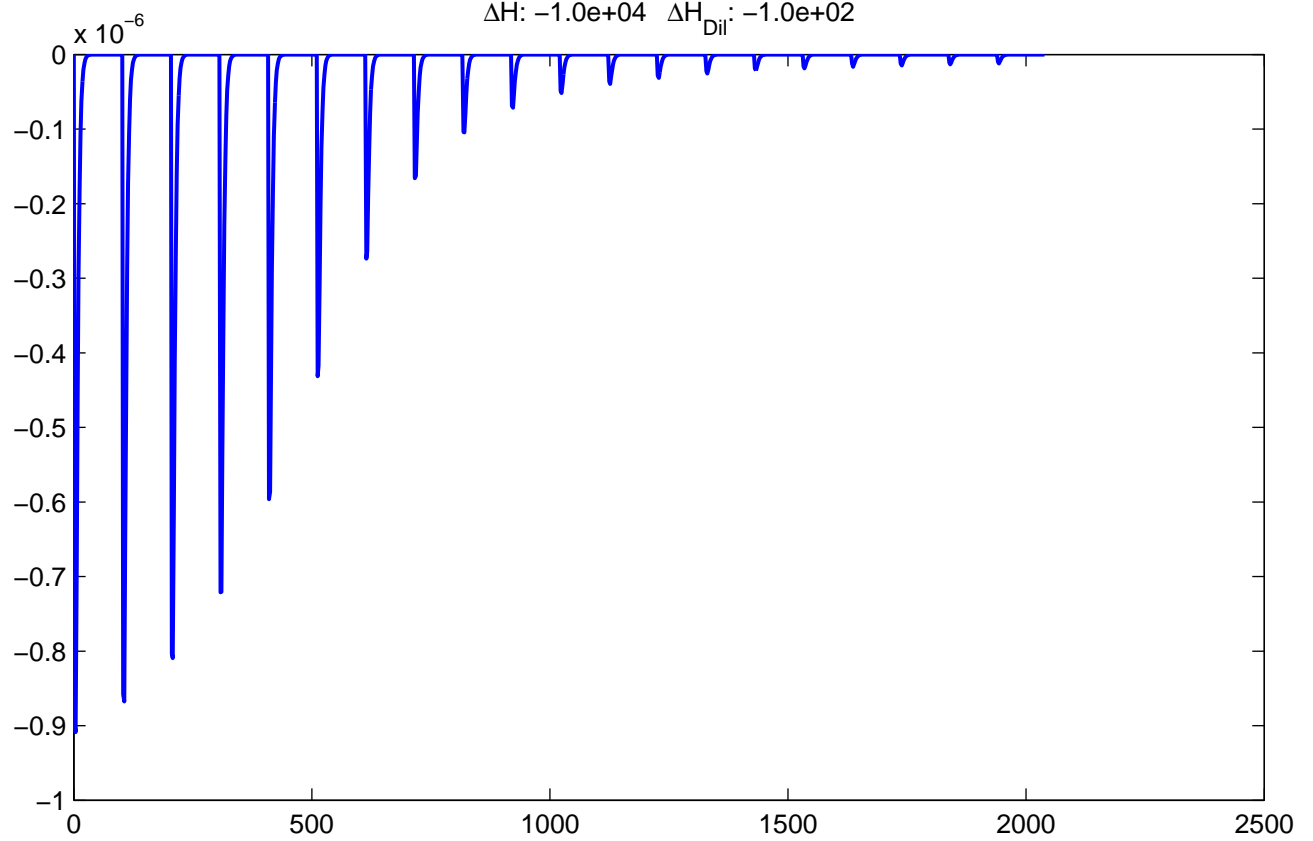

Supplement: Multimedia component 2 [file mmc2.zip › Figure_1/Kinetic_Sequential/Chromatogram.pdf]

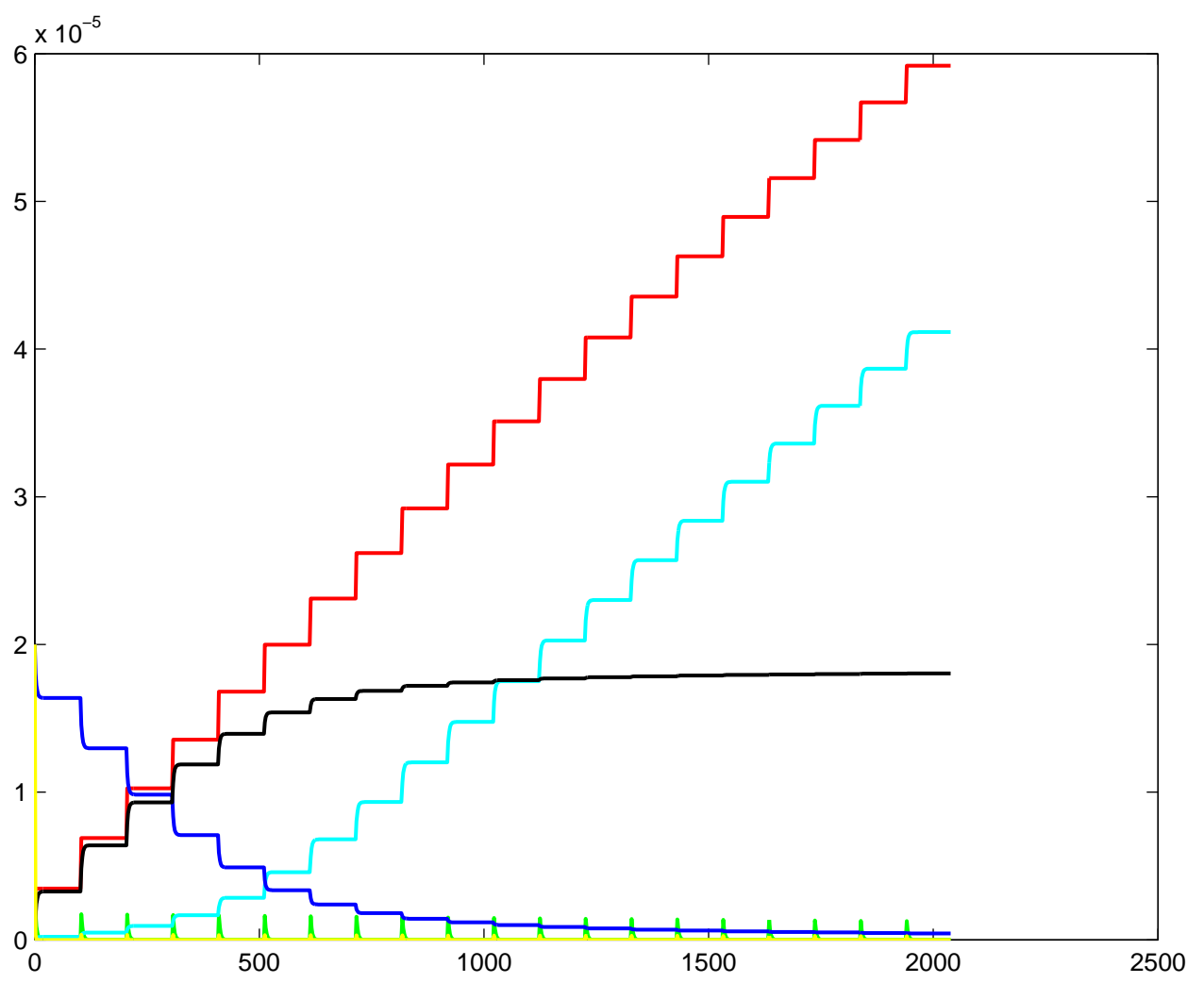

Supplement: Multimedia component 2 [file mmc2.zip › Figure_1/Kinetic_Sequential/Kinetics(P,PL,L).pdf]

Two state

$\tau_L$ : 3 (s)  $\tau_{\Delta H}$ : 3 (s)  $\tau_{\Delta H_{Dil}}$ : 3 (s)

$K_{eq}$ : 1.0e+06  $k_{on}$ : 1.0e+06  $k_{off}$ : 1.0e+00

$\Delta H$ : -1.0e+04  $\Delta H_{Dil}$ : -1.0e+02

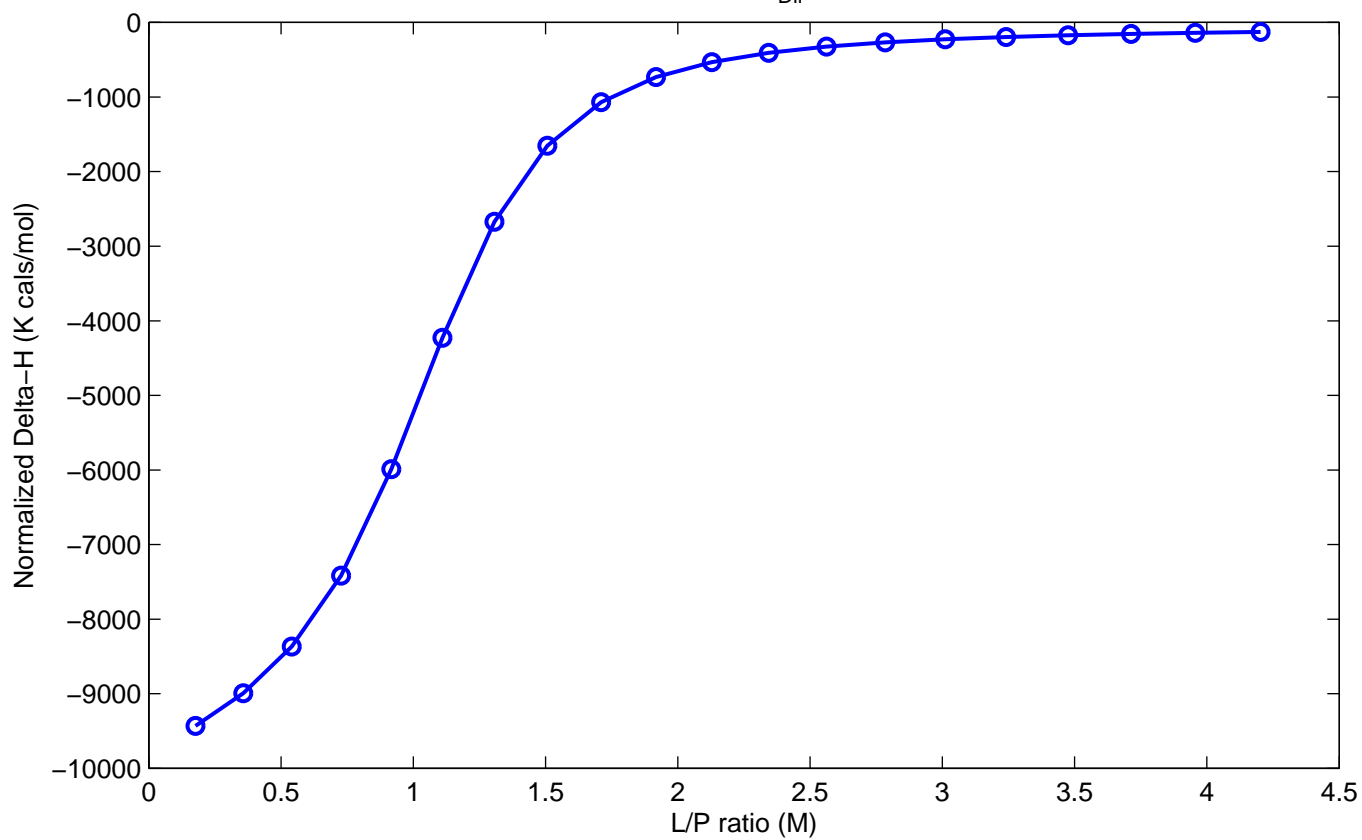

Supplement: Multimedia component 2 [file mmc2.zip › Figure_1/Kinetic_Sequential/Processed_data.pdf]

Two state

$\tau_L: 3 \text{ (s)}$   $\tau_{\Delta H}: 3 \text{ (s)}$   $\tau_{\Delta H_{Dil}}: 3 \text{ (s)}$

$K_{eq}: 1.0e+06$   $k_{on}: 1.0e+06$   $k_{off}: 1.0e+00$

$\Delta H: -1.0e+04$   $\Delta H_{Dil}: -1.0e+02$

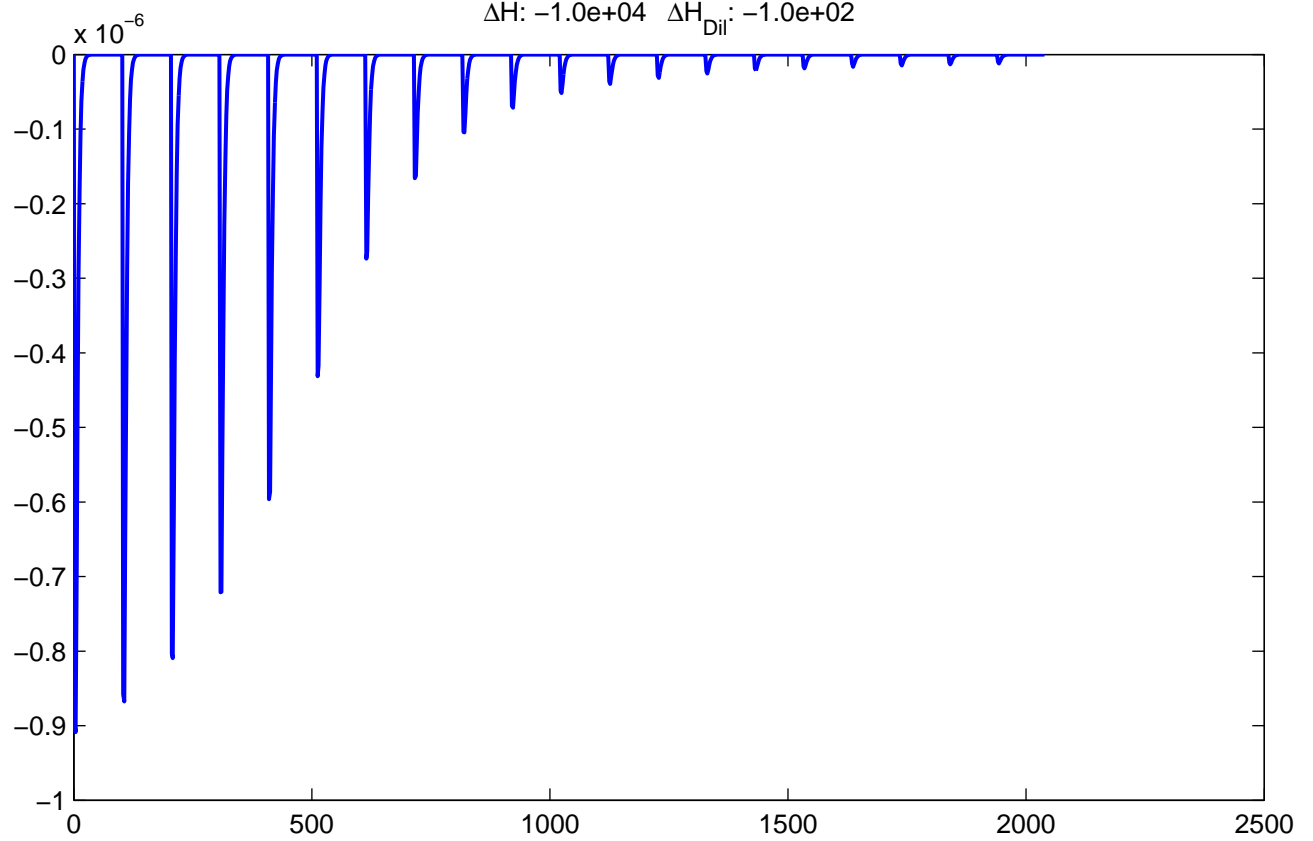

Supplement: Multimedia component 2 [file mmc2.zip › Figure_1/Laplace/Chromatogram.pdf]

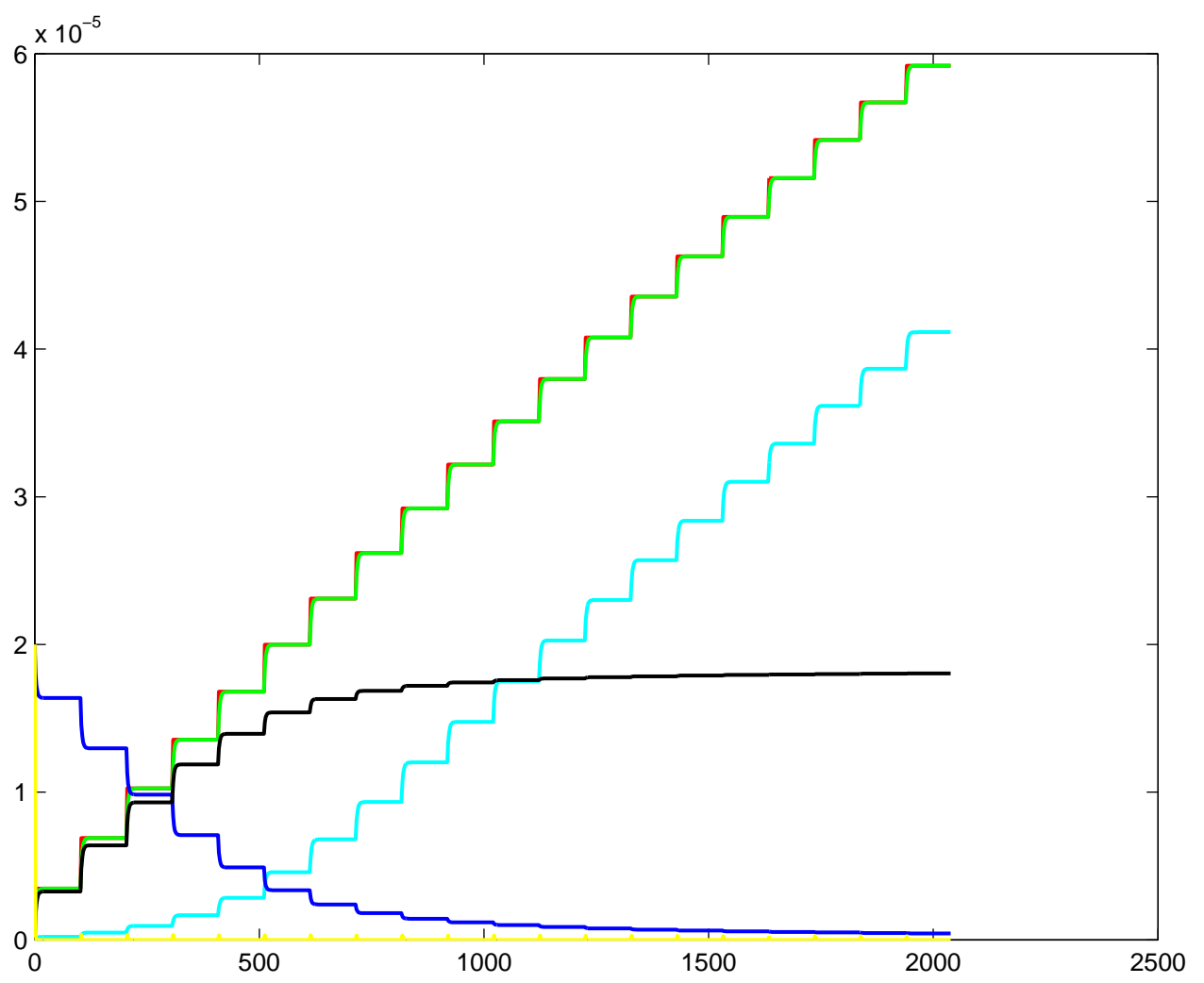

Supplement: Multimedia component 2 [file mmc2.zip › Figure_1/Laplace/Kinetics(P,PL,L).pdf]

Two state

$\tau_L$ : 3 (s)  $\tau_{\Delta H}$ : 3 (s)  $\tau_{\Delta H_{Dil}}$ : 3 (s)

$K_{eq}$ : 1.0e+06  $k_{on}$ : 1.0e+06  $k_{off}$ : 1.0e+00

$\Delta H$ : -1.0e+04  $\Delta H_{Dil}$ : -1.0e+02

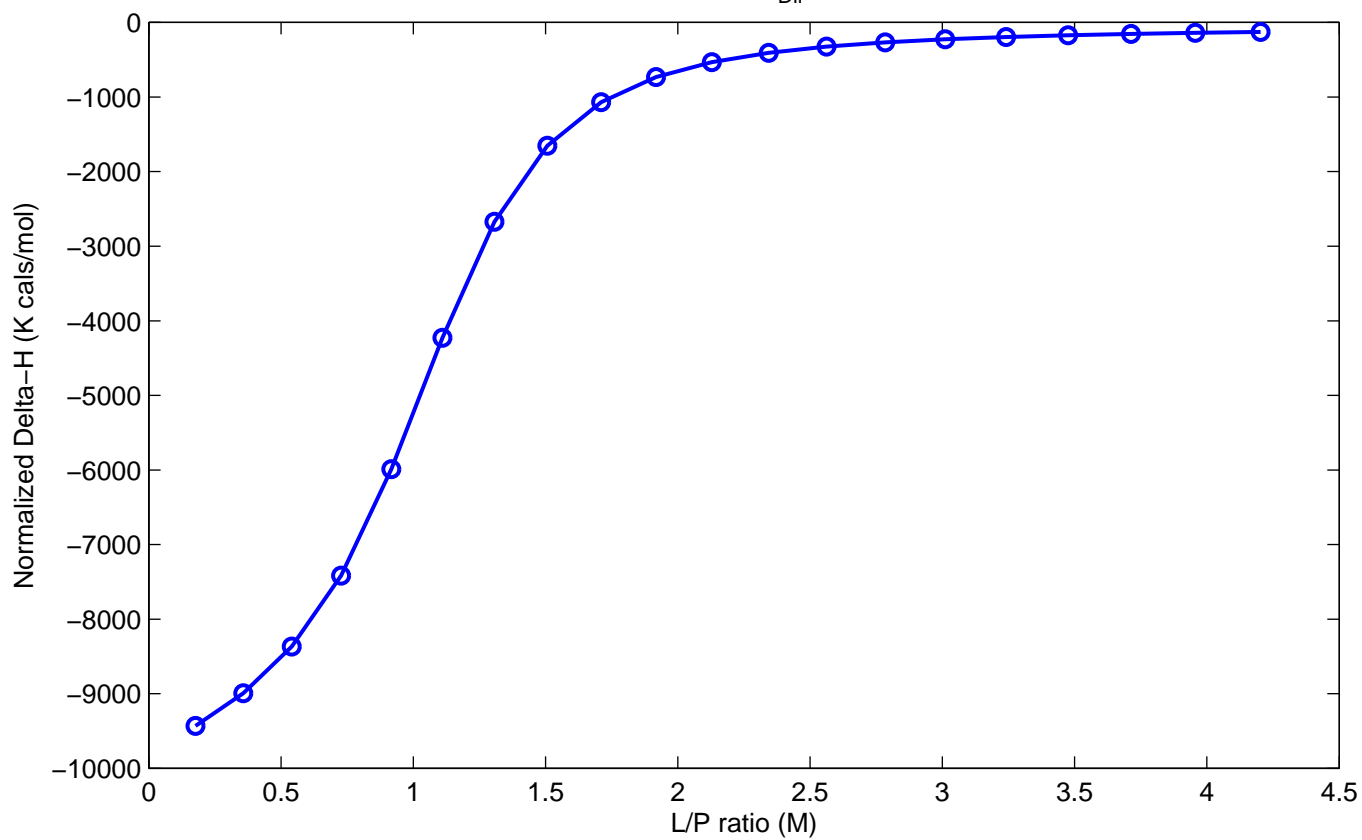

Supplement: Multimedia component 2 [file mmc2.zip › Figure_1/Laplace/Processed_data.pdf]

Two state

$\tau_L: 3 \text{ (s)}$   $\tau_{\Delta H}: 3 \text{ (s)}$   $\tau_{\Delta H_{Dil}}: 3 \text{ (s)}$

$K_{eq}: 1.0e+06$   $k_{on}: 1.0e+06$   $k_{off}: 1.0e+00$

$\Delta H: -1.0e+04$   $\Delta H_{Dil}: -1.0e+02$

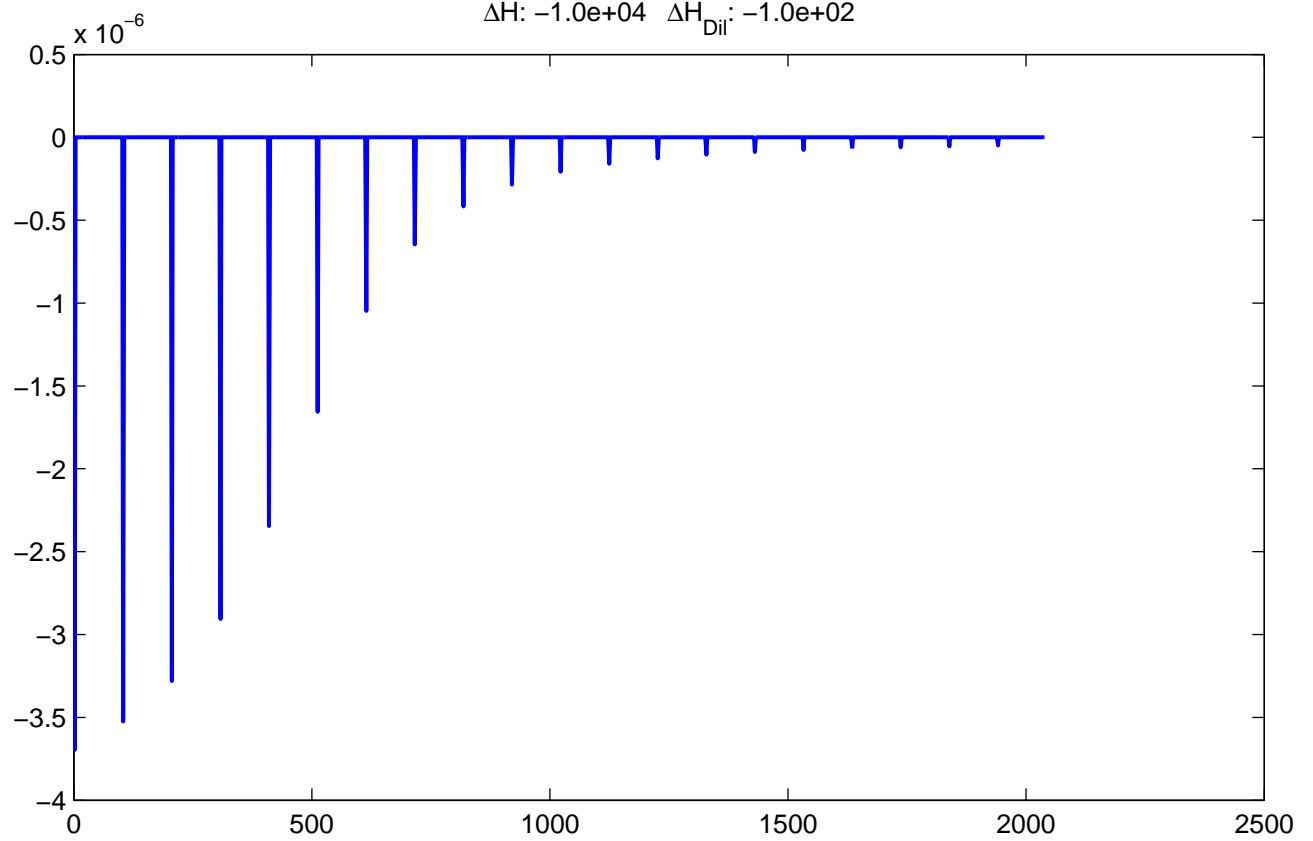

Supplement: Multimedia component 2 [file mmc2.zip › Figure_1/No_IR/Chromatogram.pdf]

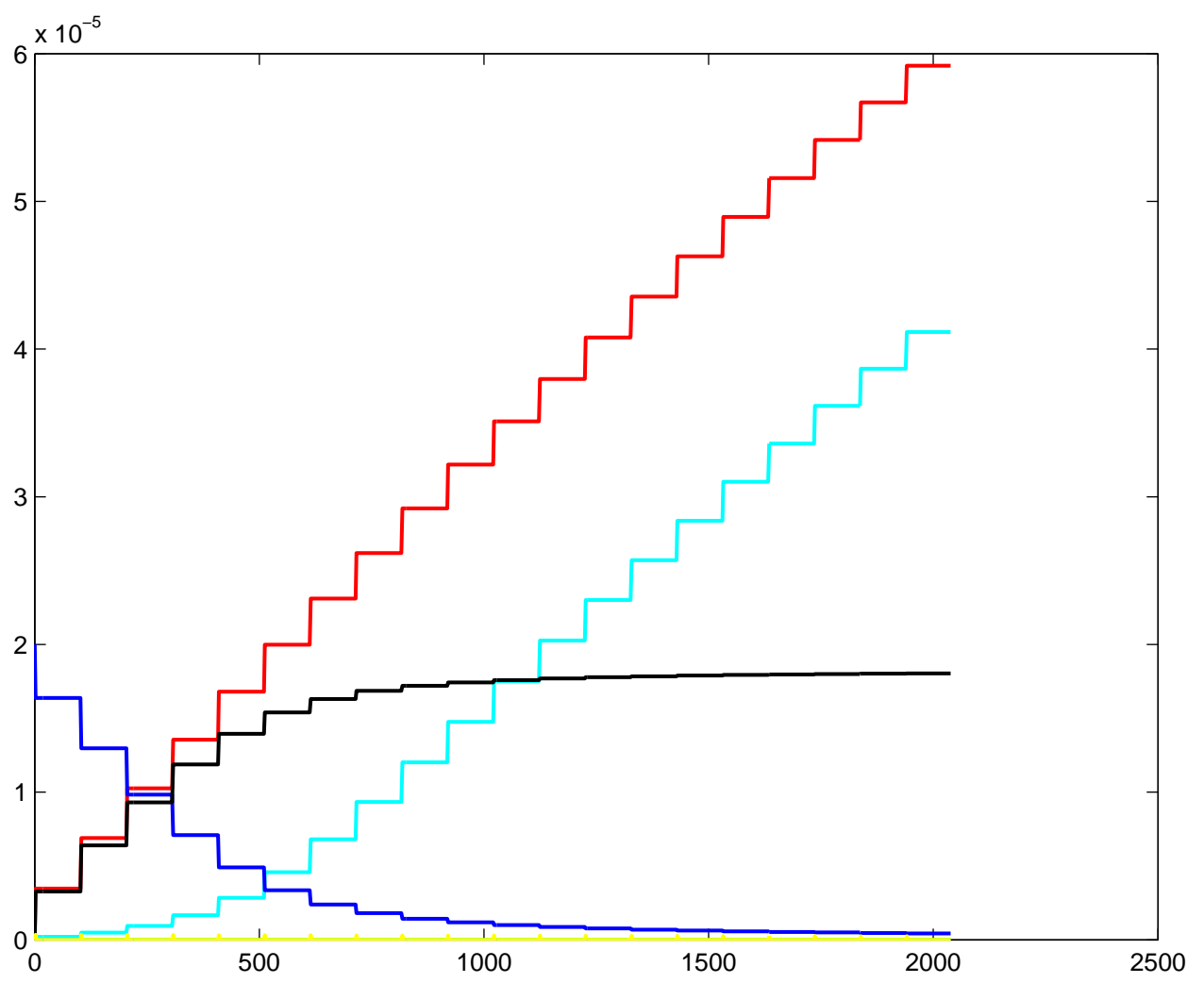

Supplement: Multimedia component 2 [file mmc2.zip › Figure_1/No_IR/Kinetics(P,PL,L).pdf]

Two state

$\tau_L$ : 3 (s)  $\tau_{\Delta H}$ : 3 (s)  $\tau_{\Delta H_{Dil}}$ : 3 (s)

$K_{eq}$ : 1.0e+06  $k_{on}$ : 1.0e+06  $k_{off}$ : 1.0e+00

$\Delta H$ : -1.0e+04  $\Delta H_{Dil}$ : -1.0e+02

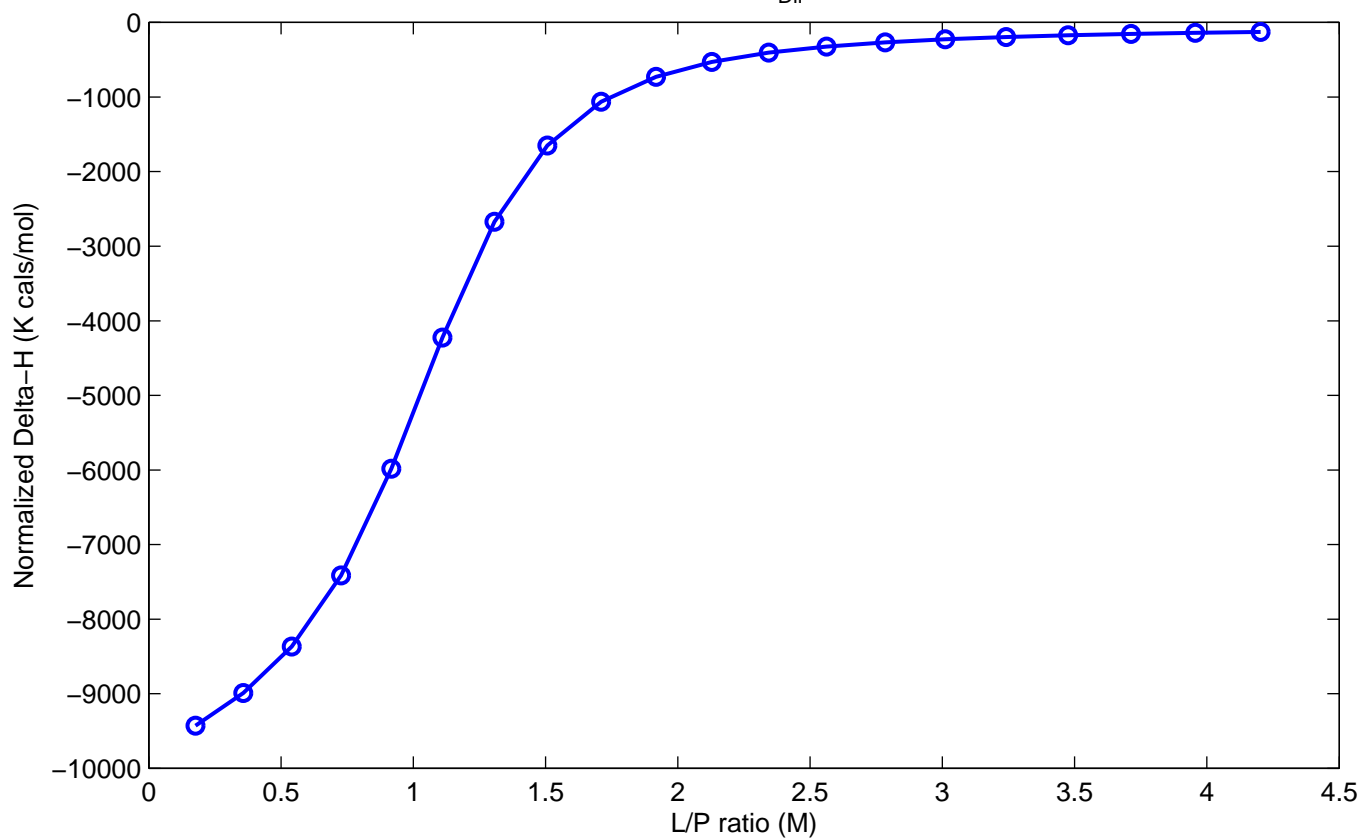

Supplement: Multimedia component 2 [file mmc2.zip › Figure_1/No_IR/Processed_data.pdf]

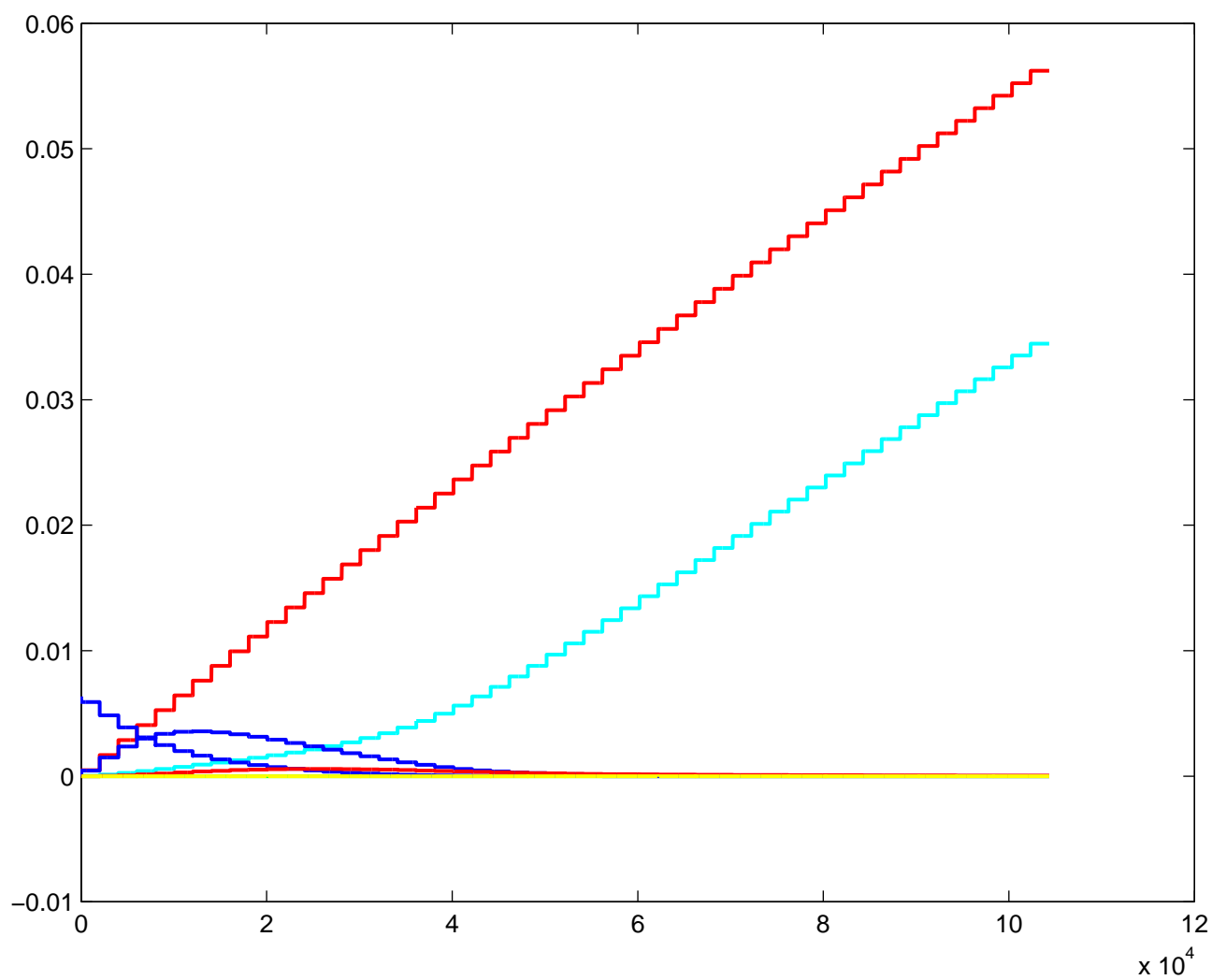

Supplement: Multimedia component 2 [file mmc2.zip › Figure_2/MNOS_Five_mixed/Time_domain/Kinetics(P,PL,L).pdf]

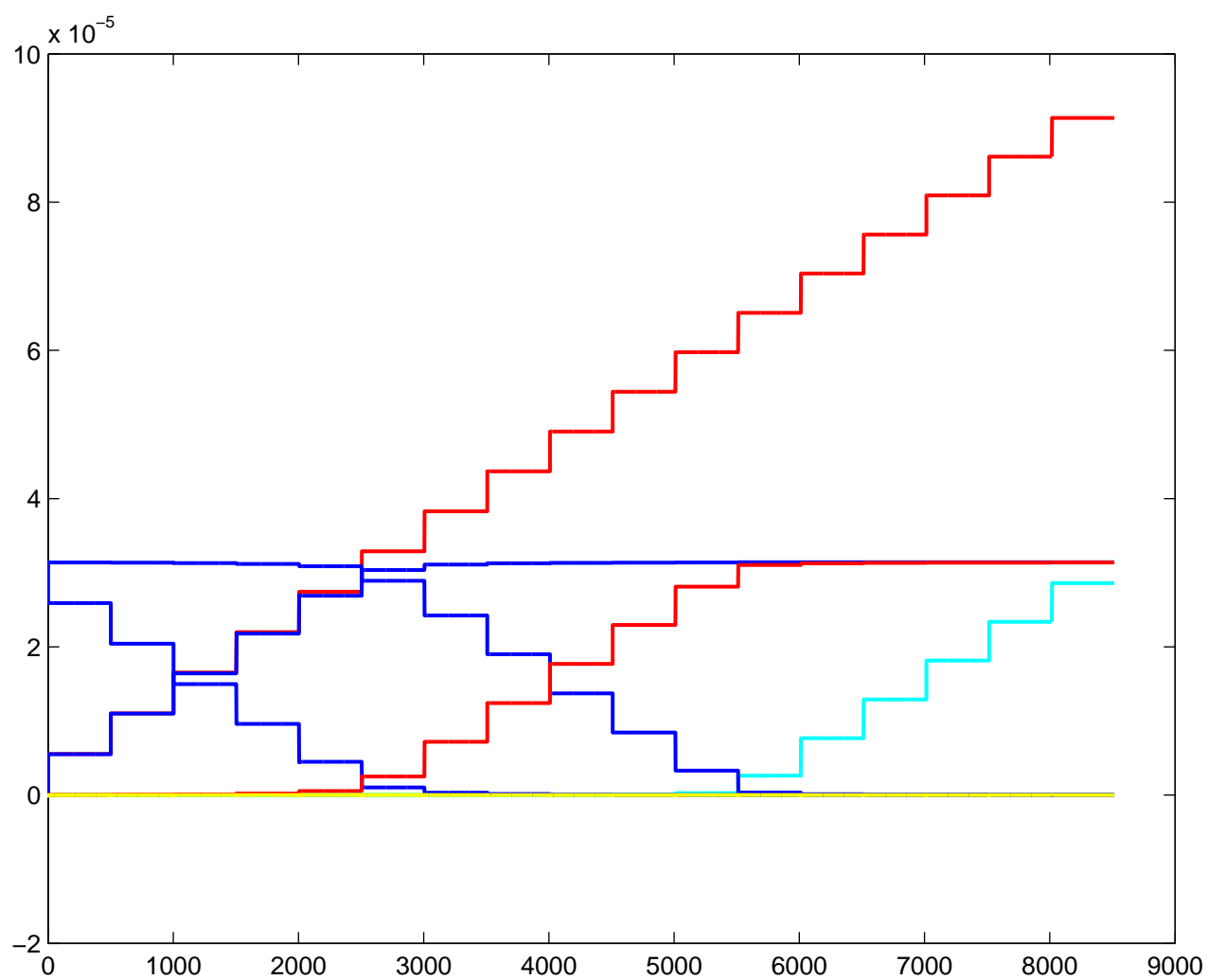

Supplement: Multimedia component 2 [file mmc2.zip › Figure_2/MN_Independent/Time_domain/Kinetics(P,PL,L).pdf]

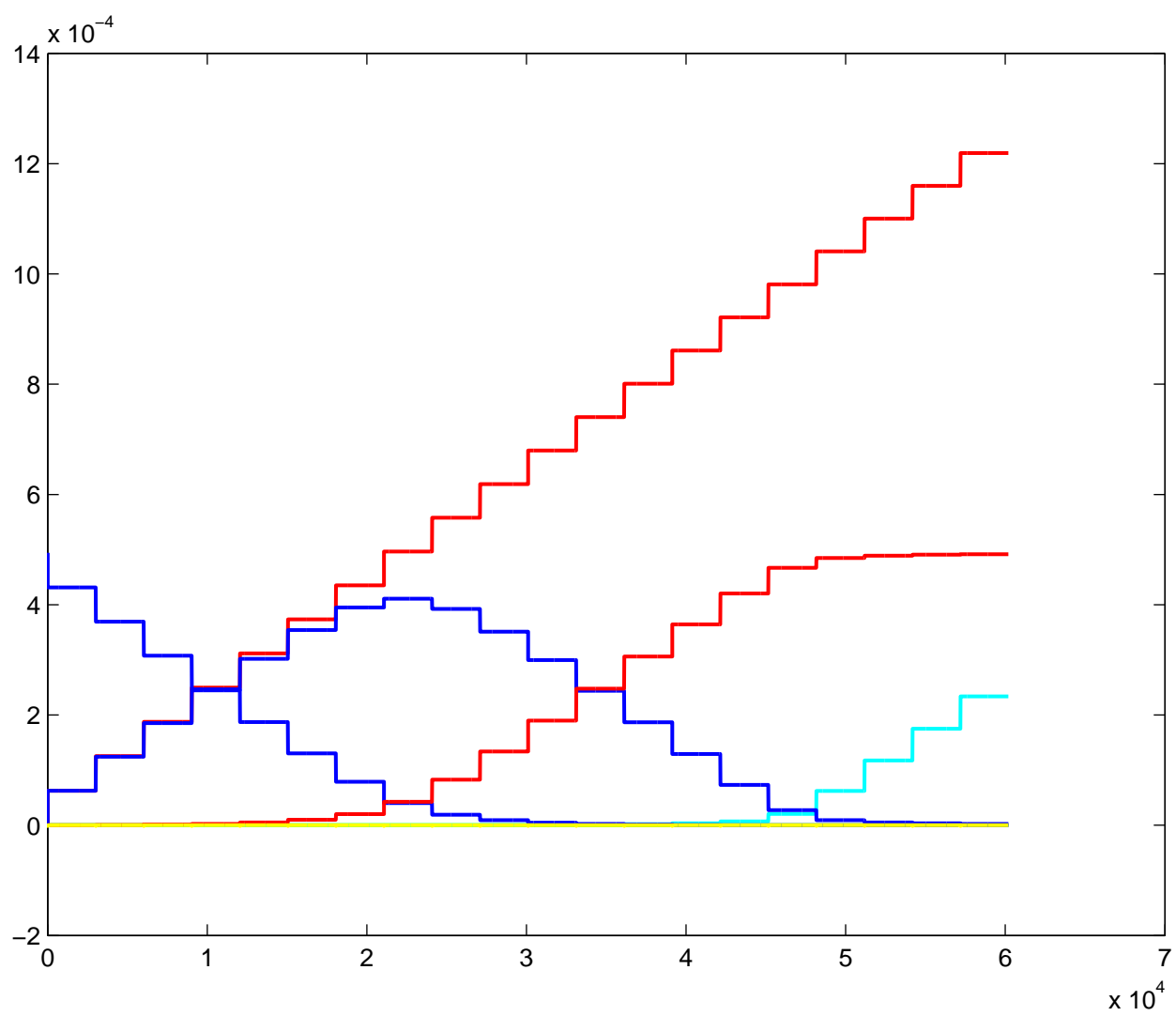

Supplement: Multimedia component 2 [file mmc2.zip › Figure_2/MN_Three_mixed/Time_domain/With_IR/Kinetics(P,PL,L).pdf]

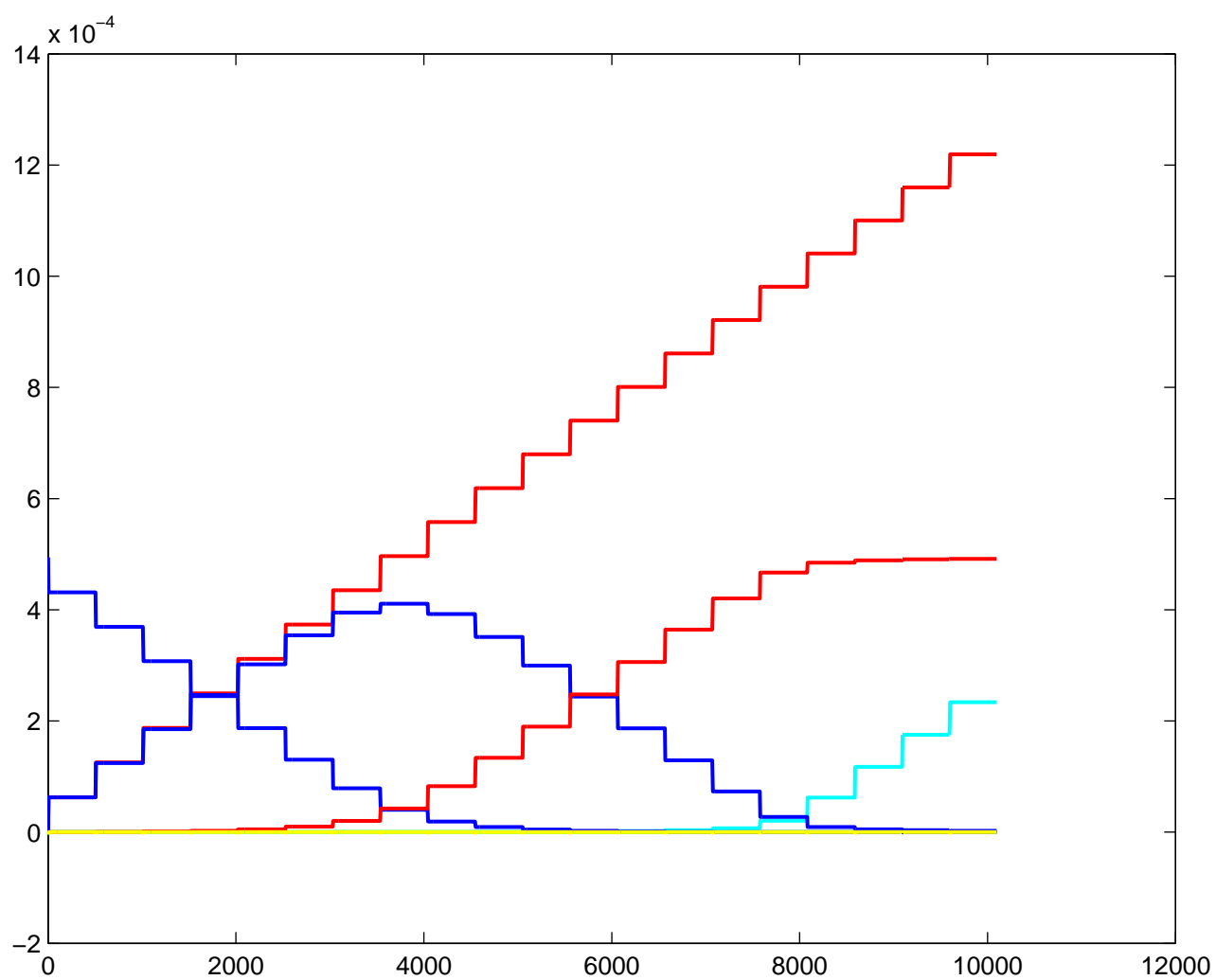

Supplement: Multimedia component 2 [file mmc2.zip › Figure_2/MN_Three_mixed/Time_domain/With_IR_1/Kinetics(P,PL,L).pdf]

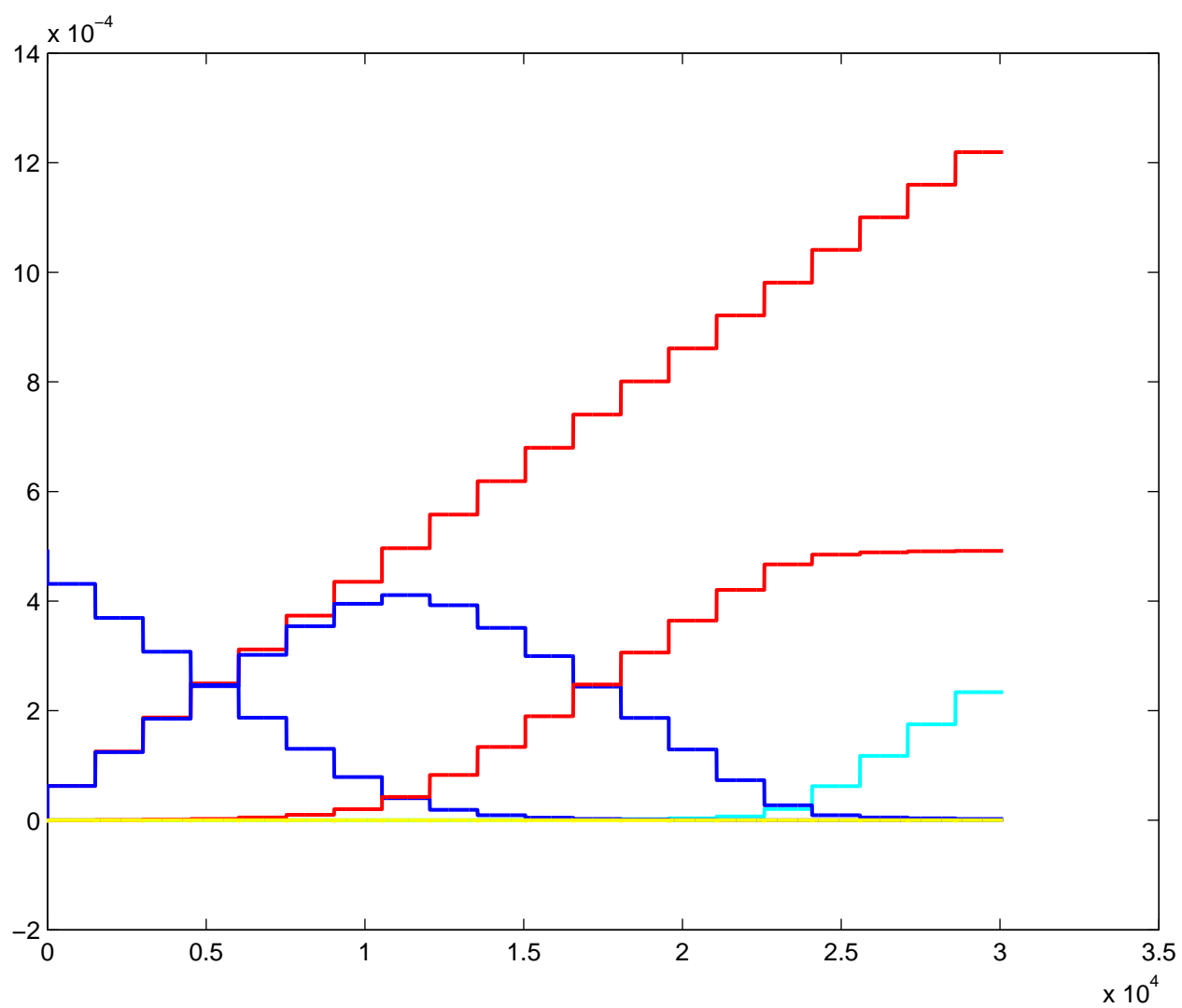

Supplement: Multimedia component 2 [file mmc2.zip › Figure_2/MN_Three_mixed/Time_domain/Without_IR/Kinetics(P,PL,L).pdf]

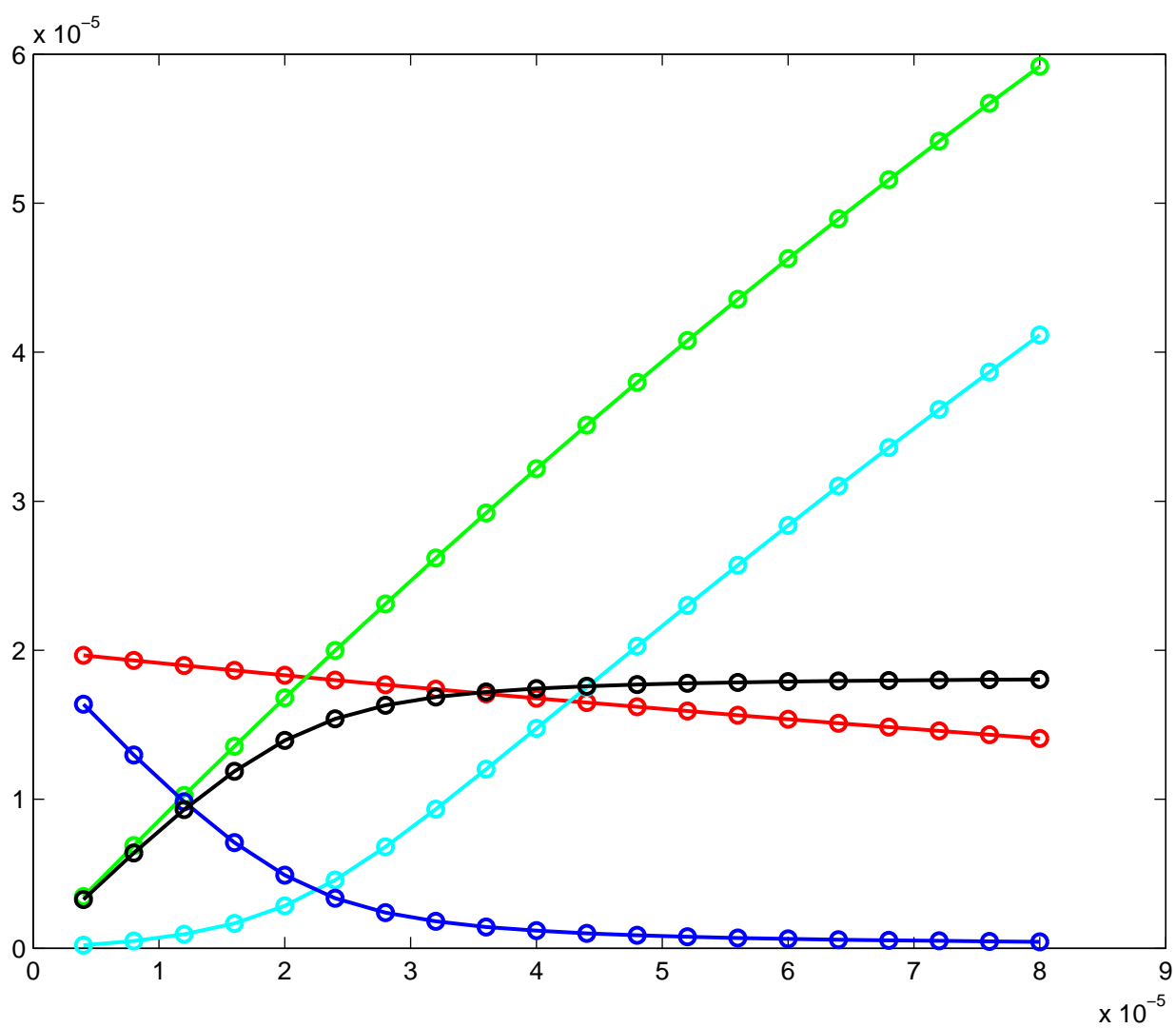

Supplement: Multimedia component 2 [file mmc2.zip › Figure_2/M_Equivalent/NDH/Numerical/Kinetics(P,PL,L).pdf]

Two states

$K_{eq}^1$ : 1.0e+06 N: -1e+04

$\Delta H$ : -1.0e+02  $\Delta H_{Dil}$ : 1.1e

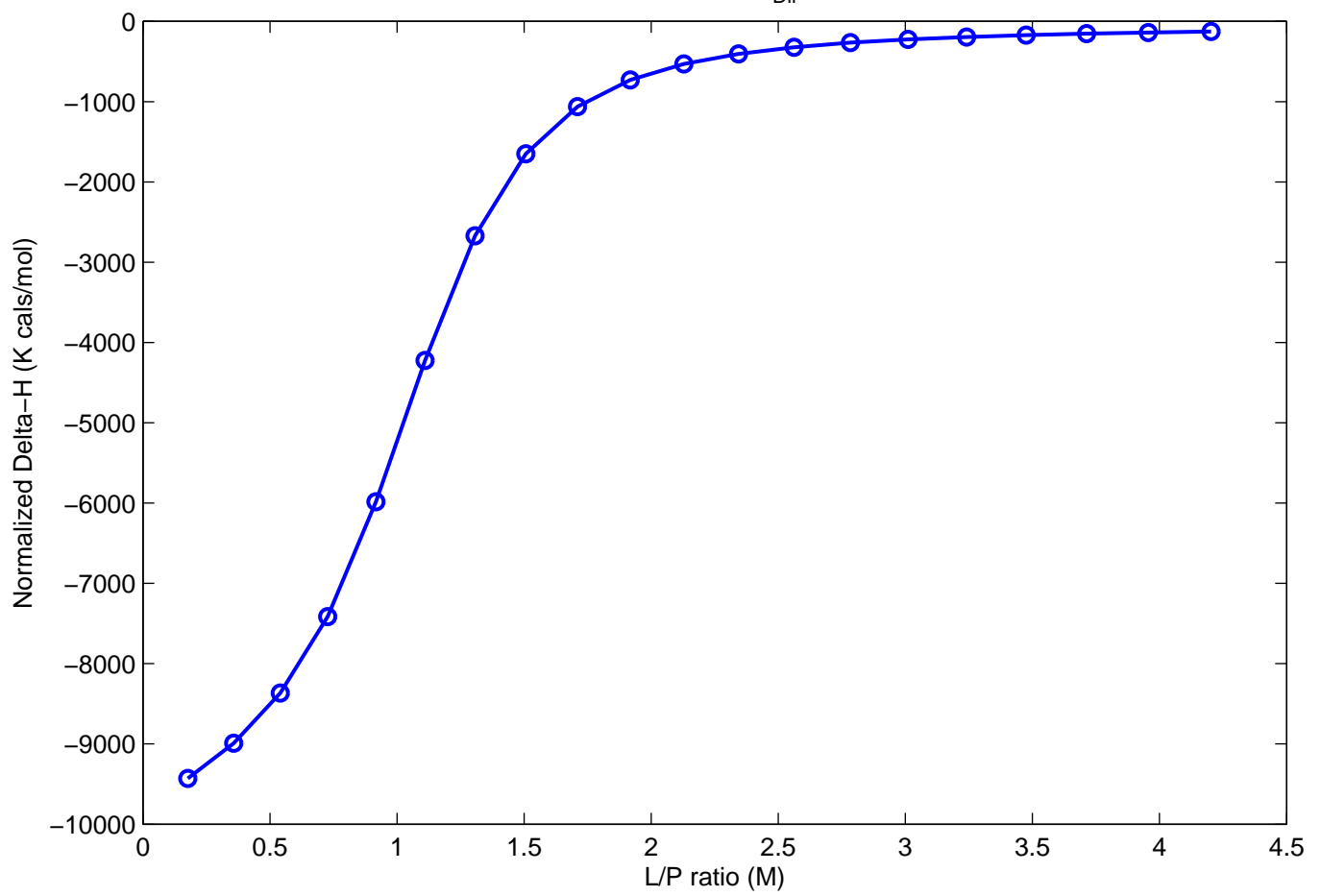

Supplement: Multimedia component 2 [file mmc2.zip › Figure_2/M_Equivalent/NDH/Numerical/NDH_plots.pdf]

Two state

$\tau_L: 3 \text{ (s)}$   $\tau_{\Delta H}: 3 \text{ (s)}$   $\tau_{\Delta H_{Dil}}: 3 \text{ (s)}$

$K_{eq}: 1.0e+06$   $k_{on}: 1.0e+06$   $k_{off}: 1.0e+00$

$\Delta H: -1.0e+04$   $\Delta H_{Dil}: -1.0e+02$

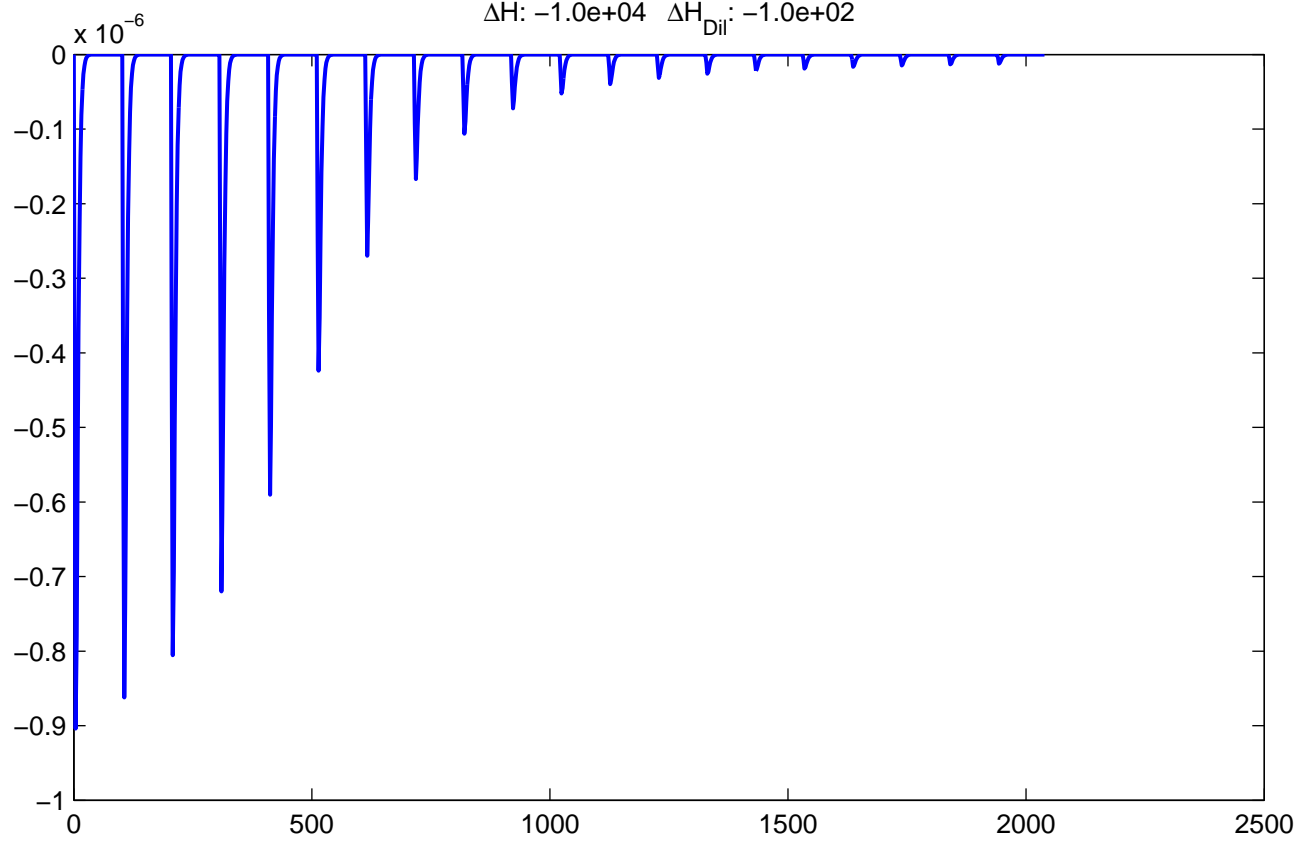

Supplement: Multimedia component 2 [file mmc2.zip › Figure_2/M_Equivalent/Time_domain/With_IR/Chromatogram.pdf]

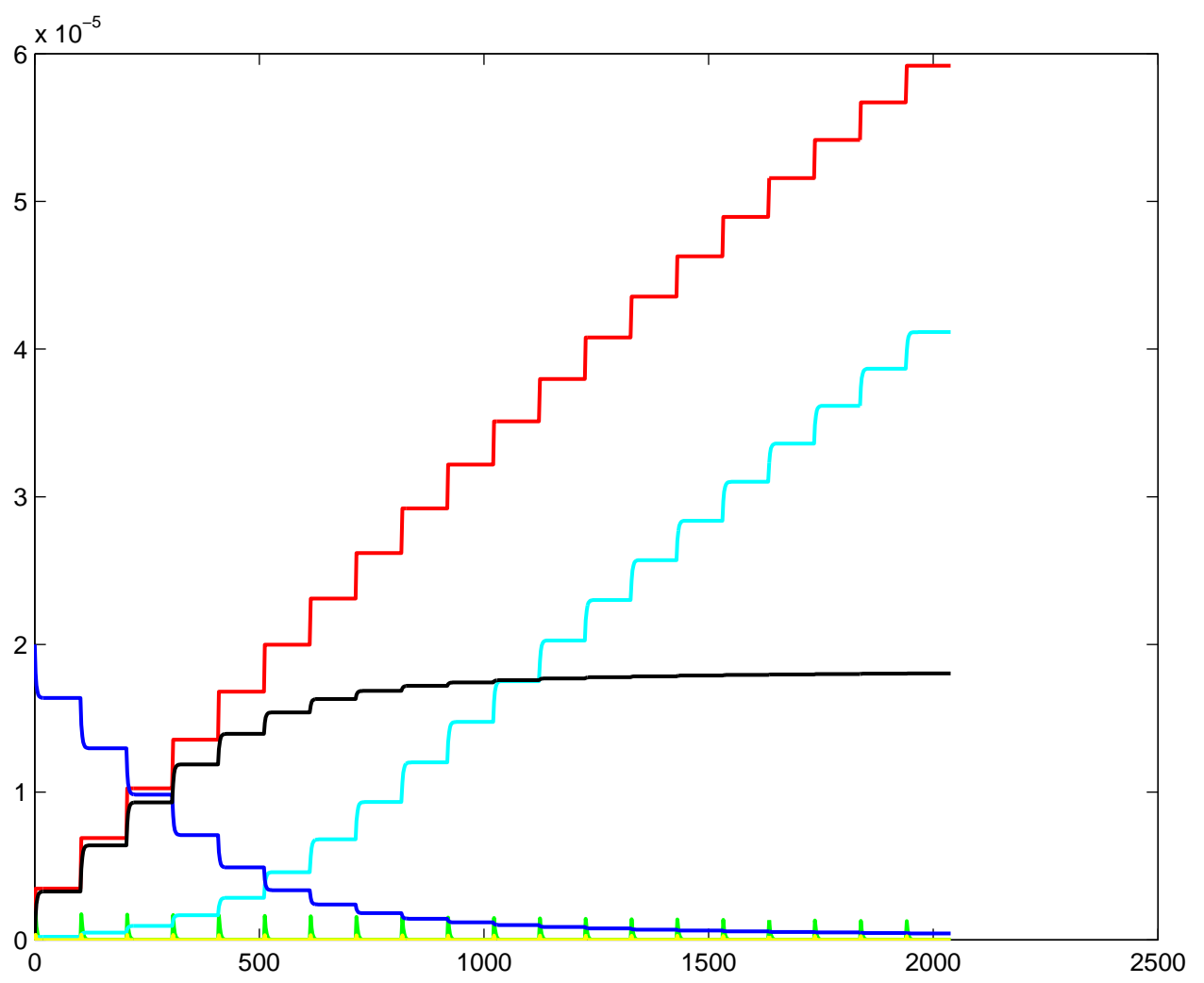

Supplement: Multimedia component 2 [file mmc2.zip › Figure_2/M_Equivalent/Time_domain/With_IR/Kinetics(P,PL,L).pdf]

Two state

$\tau_L$ : 3 (s)  $\tau_{\Delta H}$ : 3 (s)  $\tau_{\Delta H_{Dil}}$ : 3 (s)

$K_{eq}$ : 1.0e+06  $k_{on}$ : 1.0e+06  $k_{off}$ : 1.0e+00

$\Delta H$ : -1.0e+04  $\Delta H_{Dil}$ : -1.0e+02

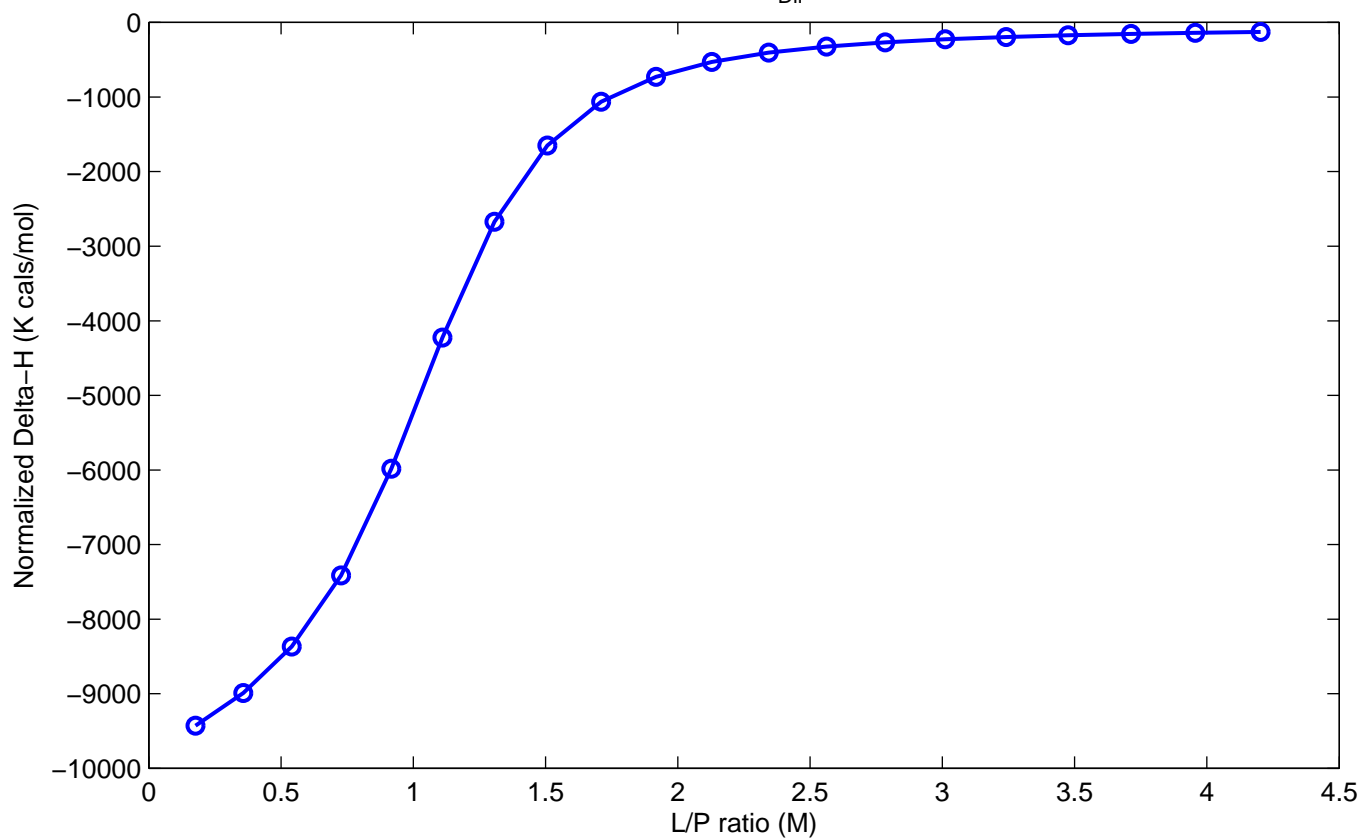

Supplement: Multimedia component 2 [file mmc2.zip › Figure_2/M_Equivalent/Time_domain/With_IR/Processed_data.pdf]

# Two state

$\tau_L : 0 \text{ (s)}$   $\tau_{\Delta H} : 0 \text{ (s)}$   $\tau_{\Delta H_{Dil}} : 0 \text{ (s)}$

$K_{eq} : 1.0e+06$   $k_{on} : 1.0e+06$   $k_{off} : 1.0e+00$

$\Delta H : -1.0e+04$   $\Delta H_{Dil} : -1.0e+02$

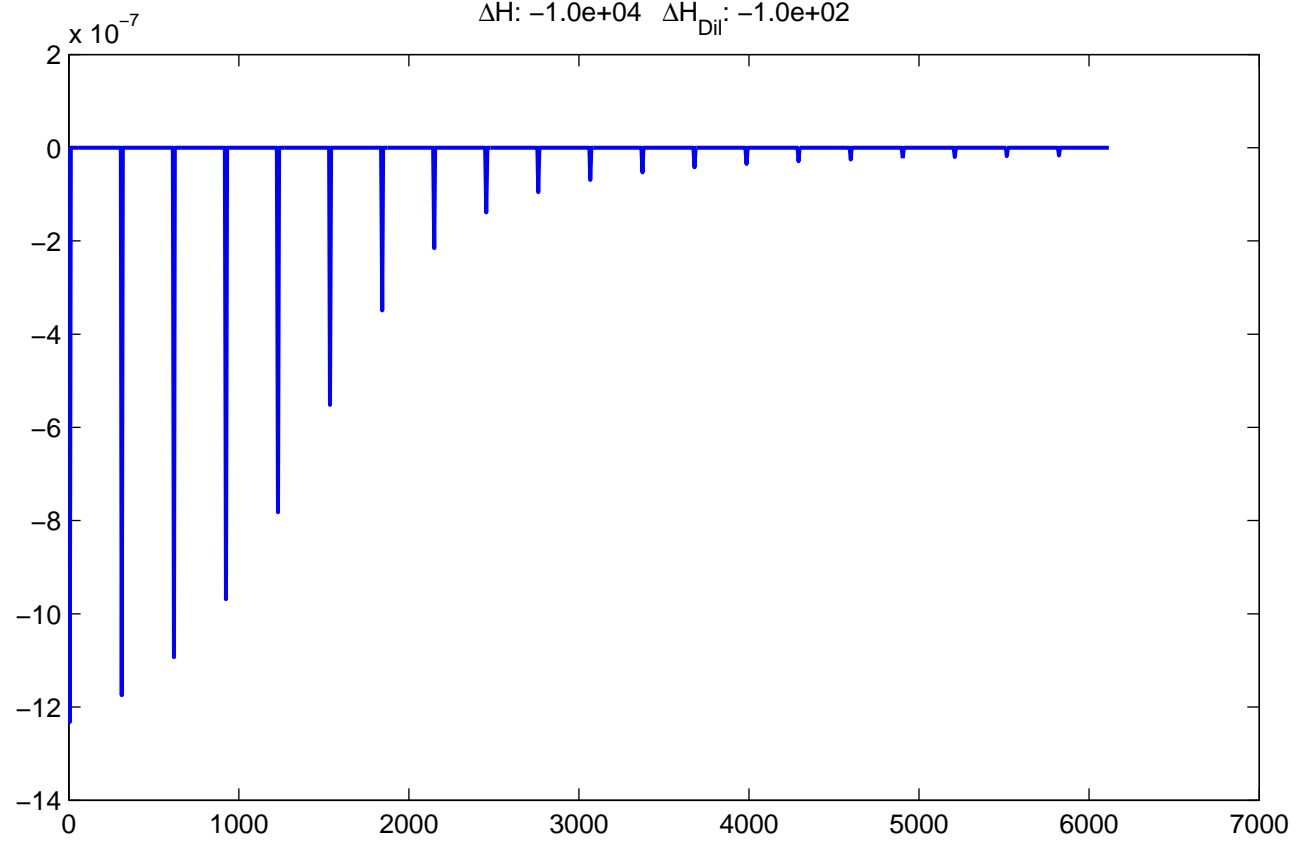

Supplement: Multimedia component 2 [file mmc2.zip › Figure_2/M_Equivalent/Time_domain/Without_IR/Chromatogram.pdf]

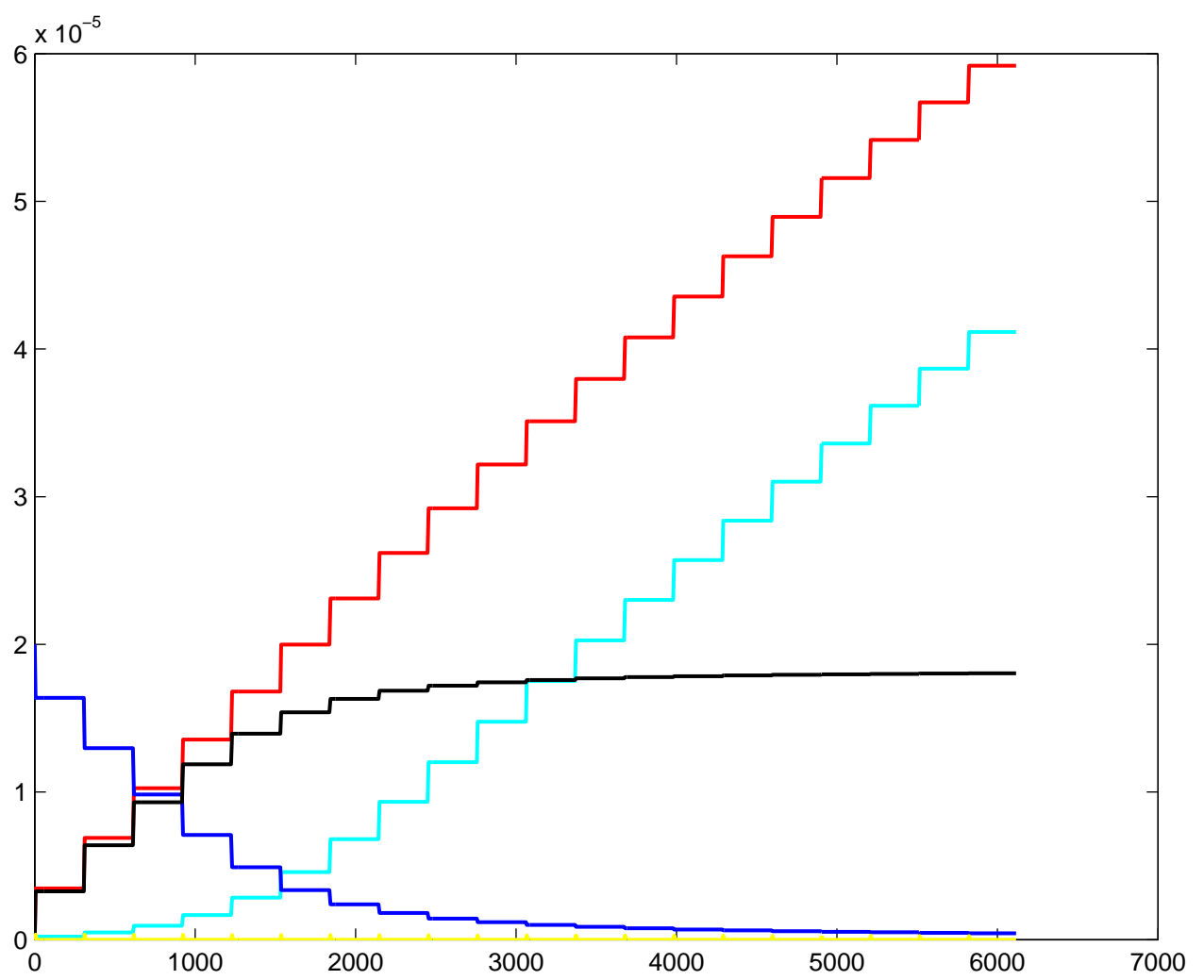

Supplement: Multimedia component 2 [file mmc2.zip › Figure_2/M_Equivalent/Time_domain/Without_IR/Kinetics(P,PL,L).pdf]

Two state

$\tau_L$ : 0 (s)  $\tau_{\Delta H}$ : 0 (s)  $\tau_{\Delta H_{Dil}}$ : 0 (s)

$K_{eq}$ : 1.0e+06  $k_{on}$ : 1.0e+06  $k_{off}$ : 1.0e+00

$\Delta H$ : -1.0e+04  $\Delta H_{Dil}$ : -1.0e+02

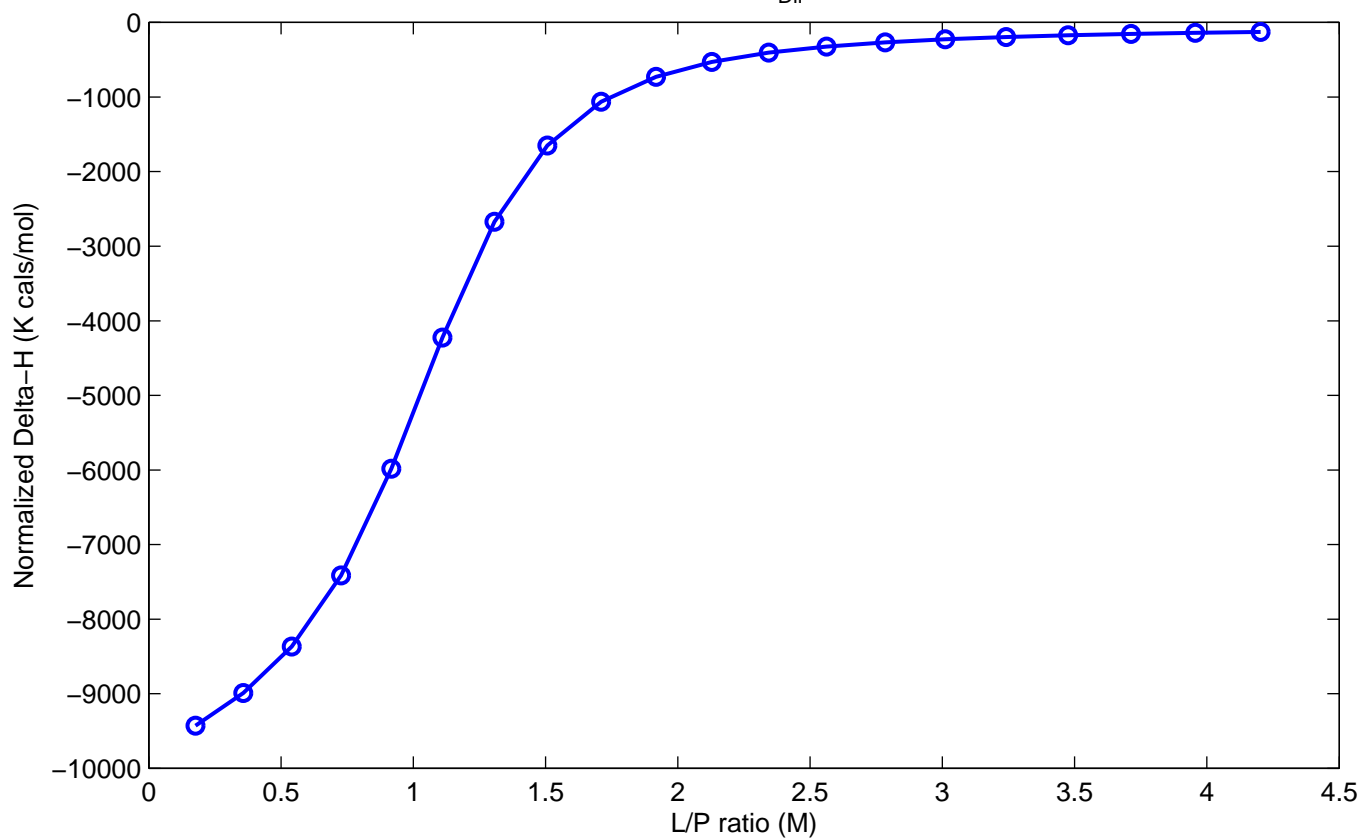

Supplement: Multimedia component 2 [file mmc2.zip › Figure_2/M_Equivalent/Time_domain/Without_IR/Processed_data.pdf]

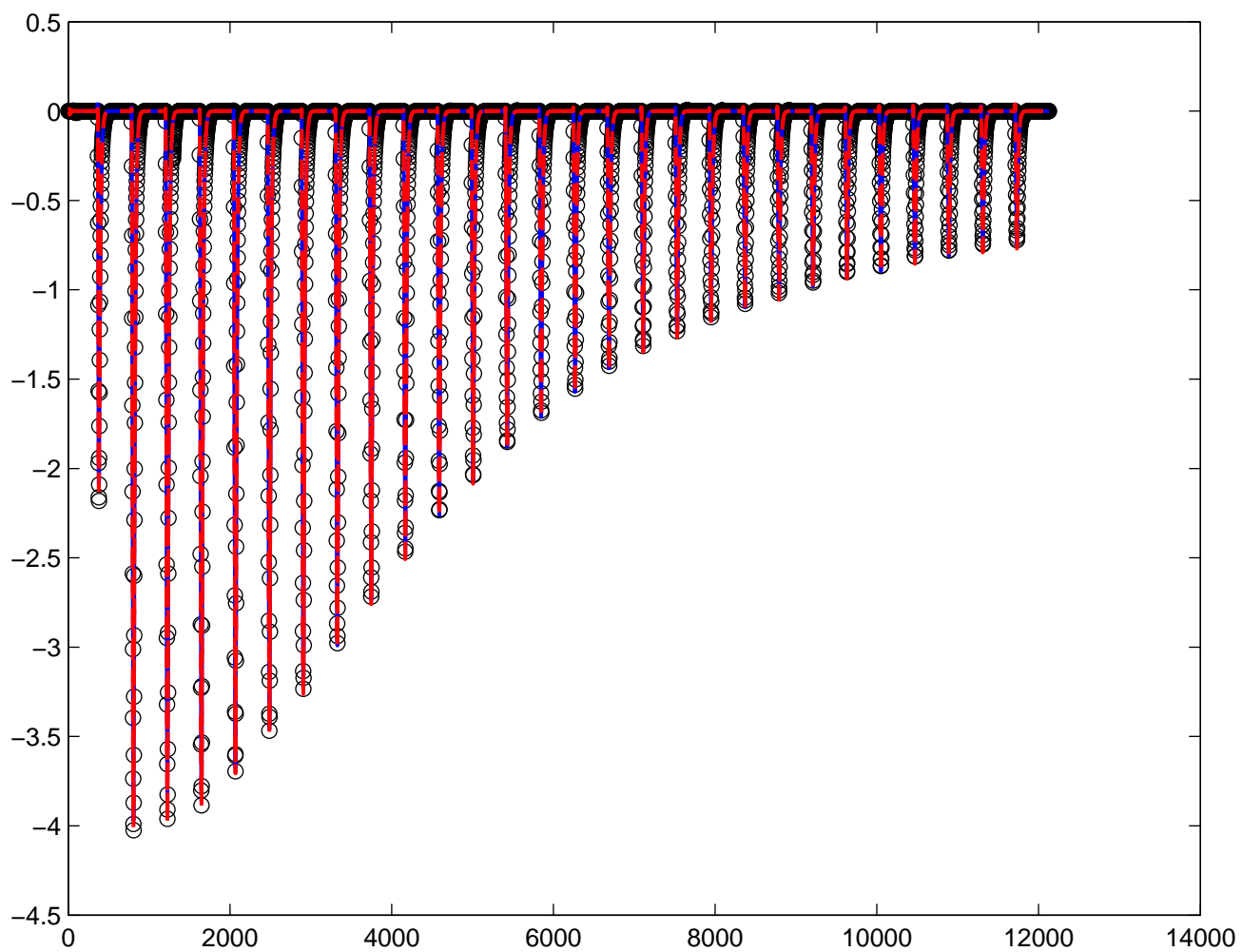

Supplement: Multimedia component 2 [file mmc2.zip › Figure_3/BCLXL/Final_plot.pdf]

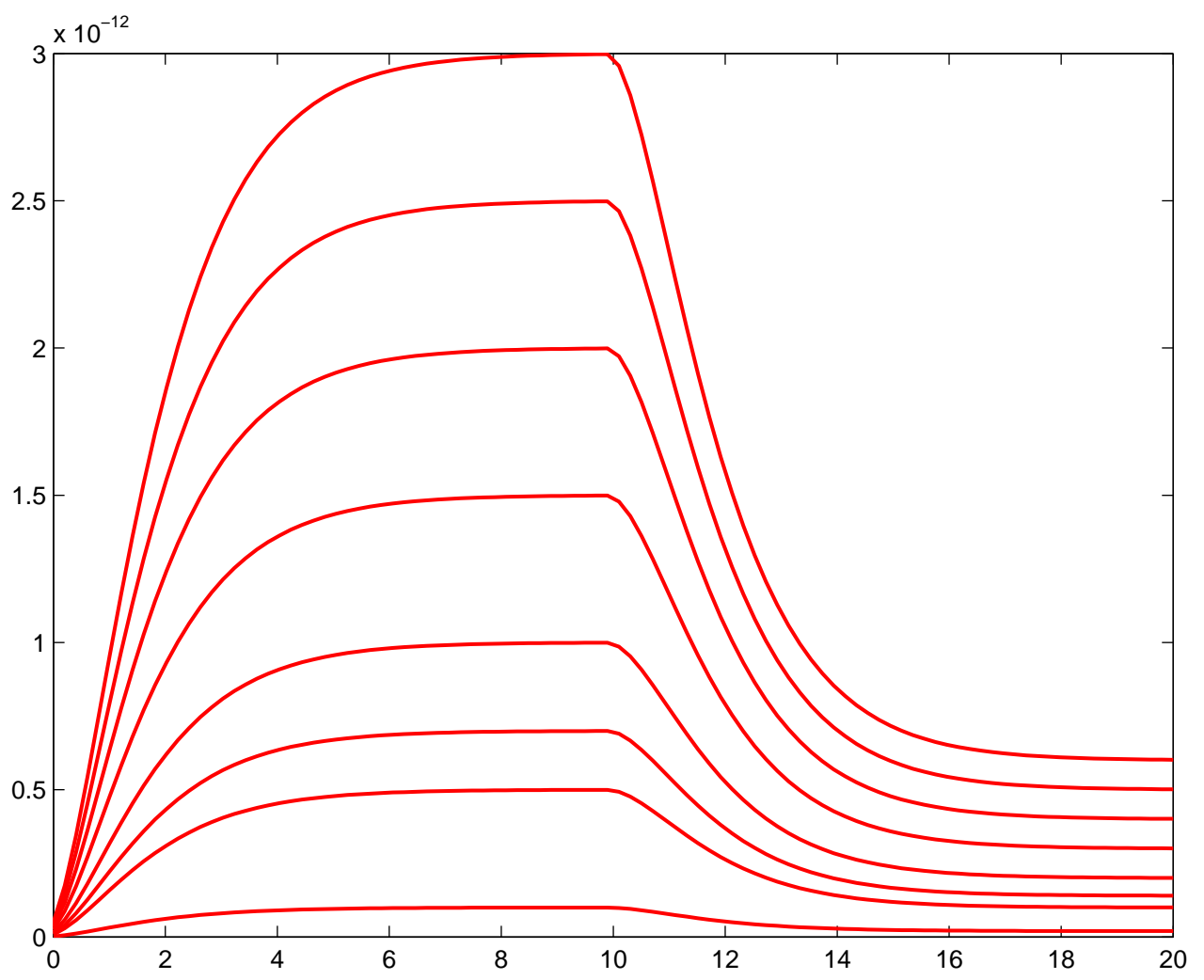

Supplement: Multimedia component 2 [file mmc2.zip › Figure_4/Leakage/Kinetics(P,PL,L).pdf]

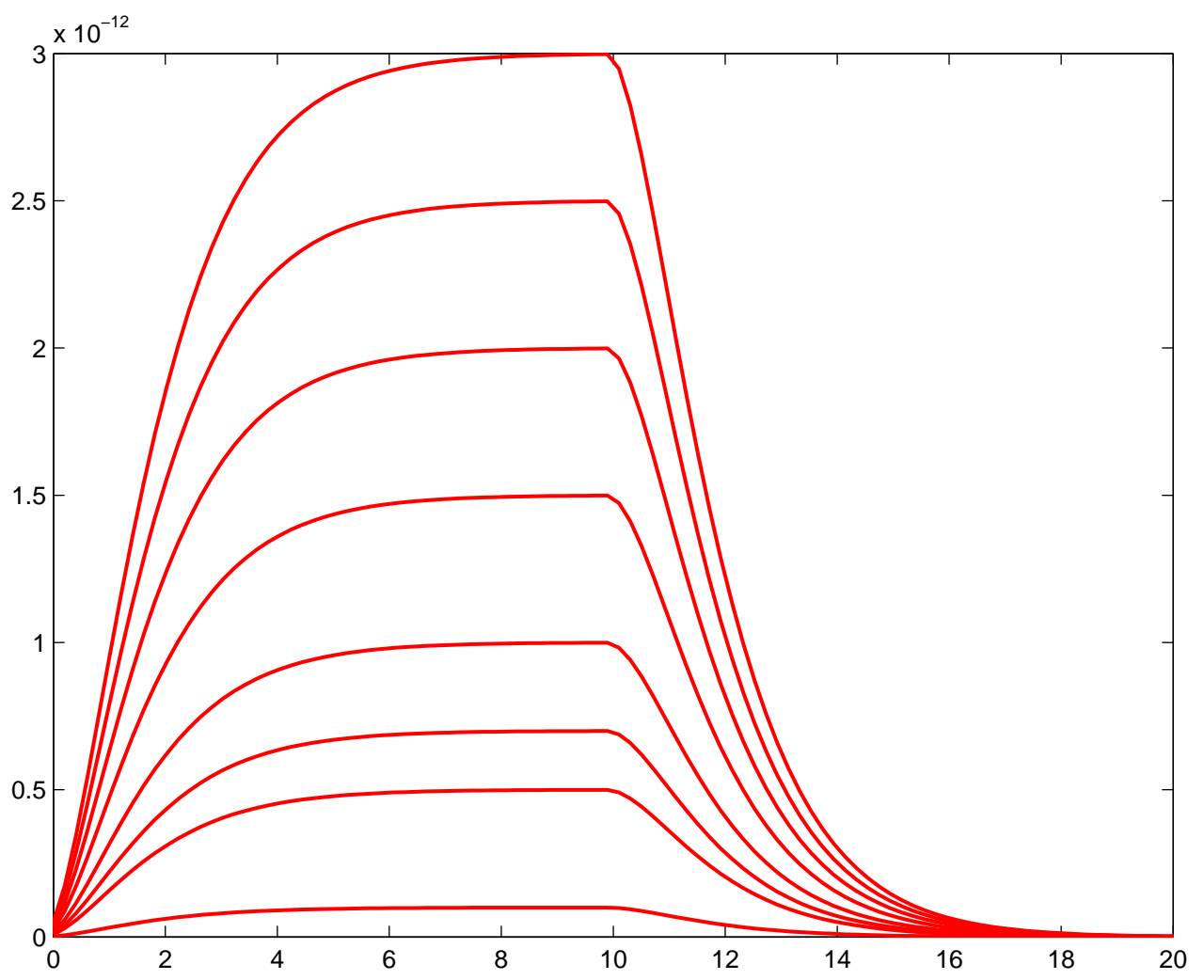

Supplement: Multimedia component 2 [file mmc2.zip › Figure_4/No_Leakage/Kinetics(P,PL,L).pdf]

Two state

$\tau_L: 3 \text{ (s)}$   $\tau_{\Delta H}: 3 \text{ (s)}$   $\tau_{\Delta H_{Dil}}: 3 \text{ (s)}$

$K_{eq}: 1.0e+06$   $k_{on}: 1.0e+06$   $k_{off}: 1.0e+00$

$\Delta H: -1.0e+04$   $\Delta H_{Dil}: -1.0e+02$

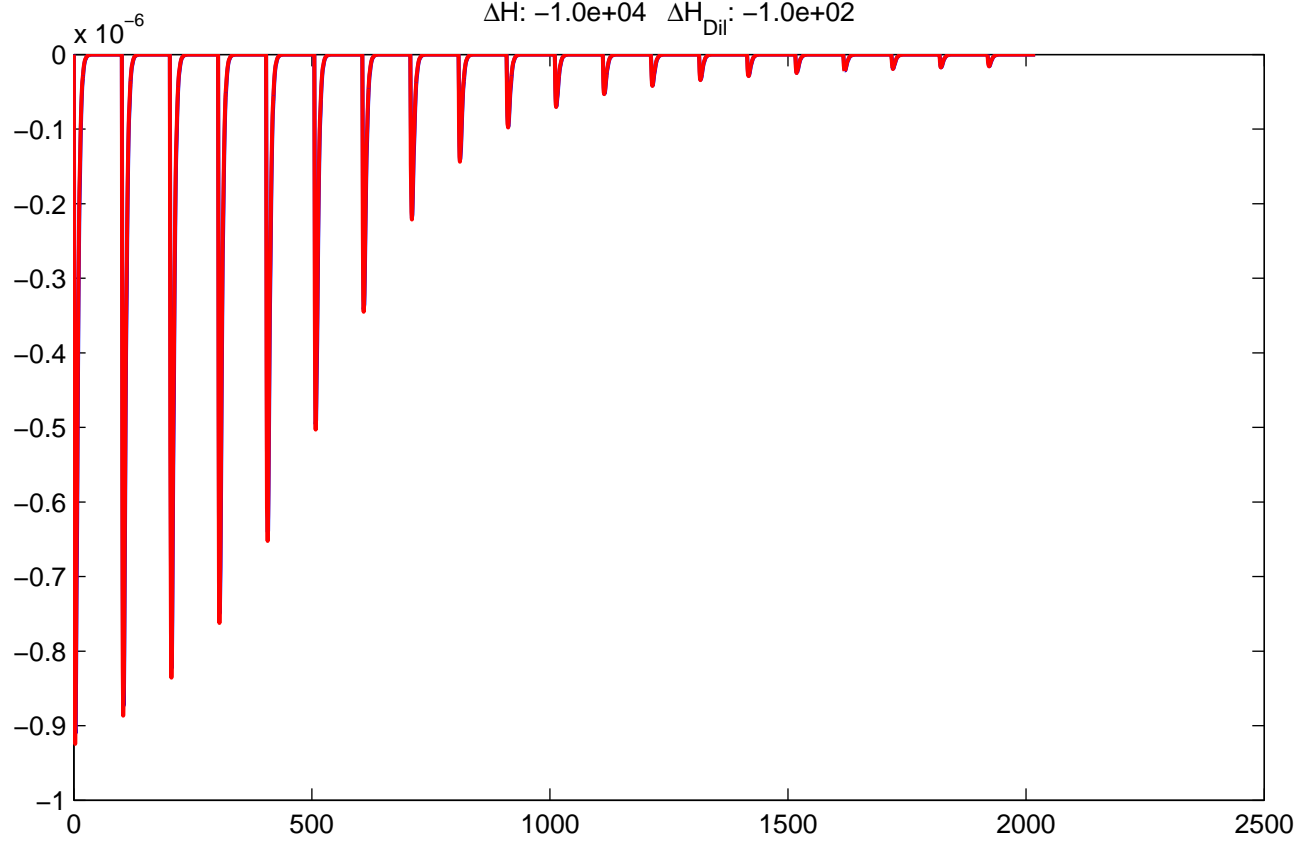

Supplement: Multimedia component 2 [file mmc2.zip › SI_Figure_S1/100_Pts/Chromatogram.pdf]

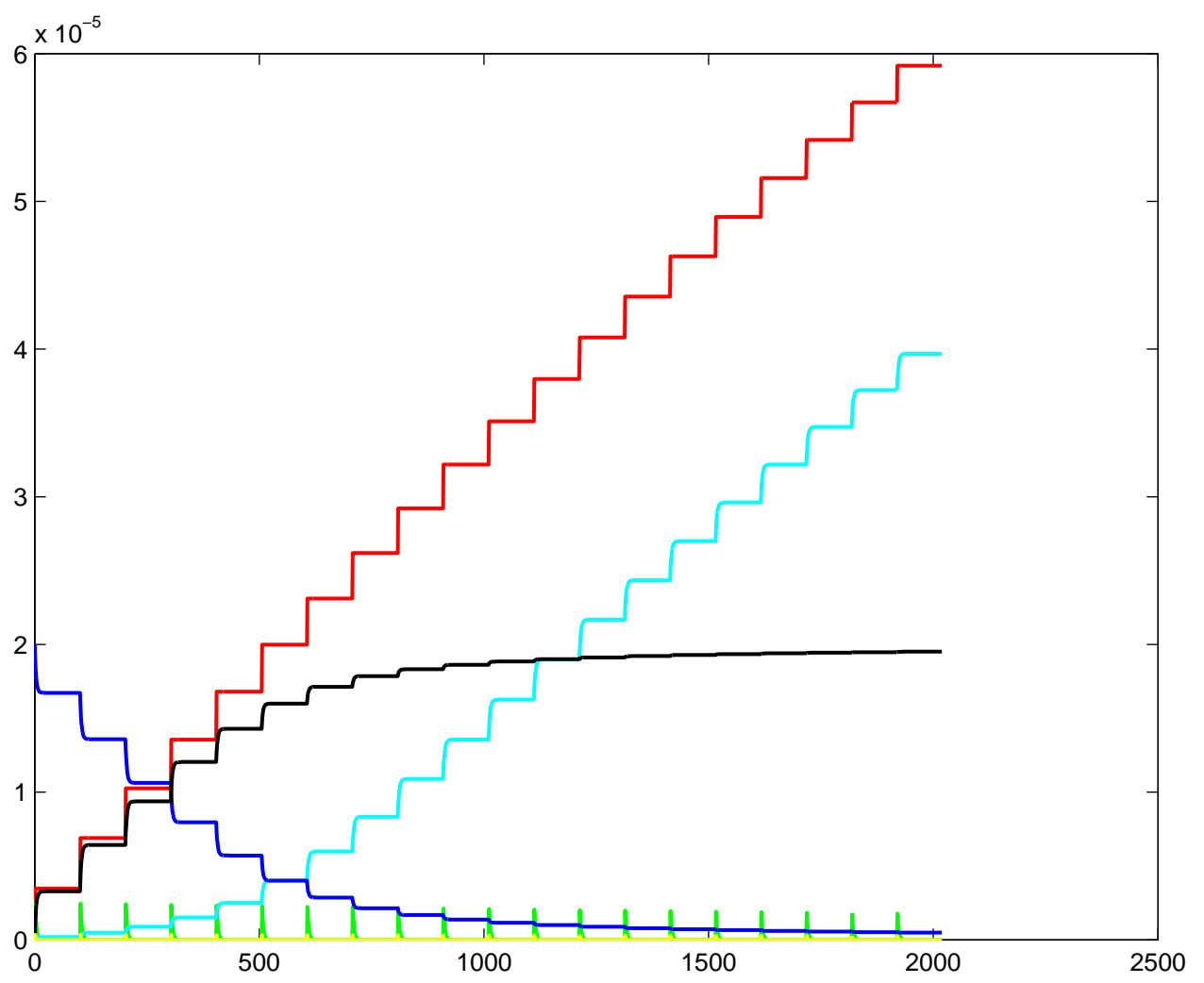

Supplement: Multimedia component 2 [file mmc2.zip › SI_Figure_S1/100_Pts/Kinetics(P,PL,L).pdf]

Two state

$\tau_L$ : 3 (s)  $\tau_{\Delta H}$ : 3 (s)  $\tau_{\Delta H_{Dil}}$ : 3 (s)

$K_{eq}$ : 1.0e+06  $k_{on}$ : 1.0e+06  $k_{off}$ : 1.0e+00

$\Delta H$ : -1.0e+04  $\Delta H_{Dil}$ : -1.0e+02

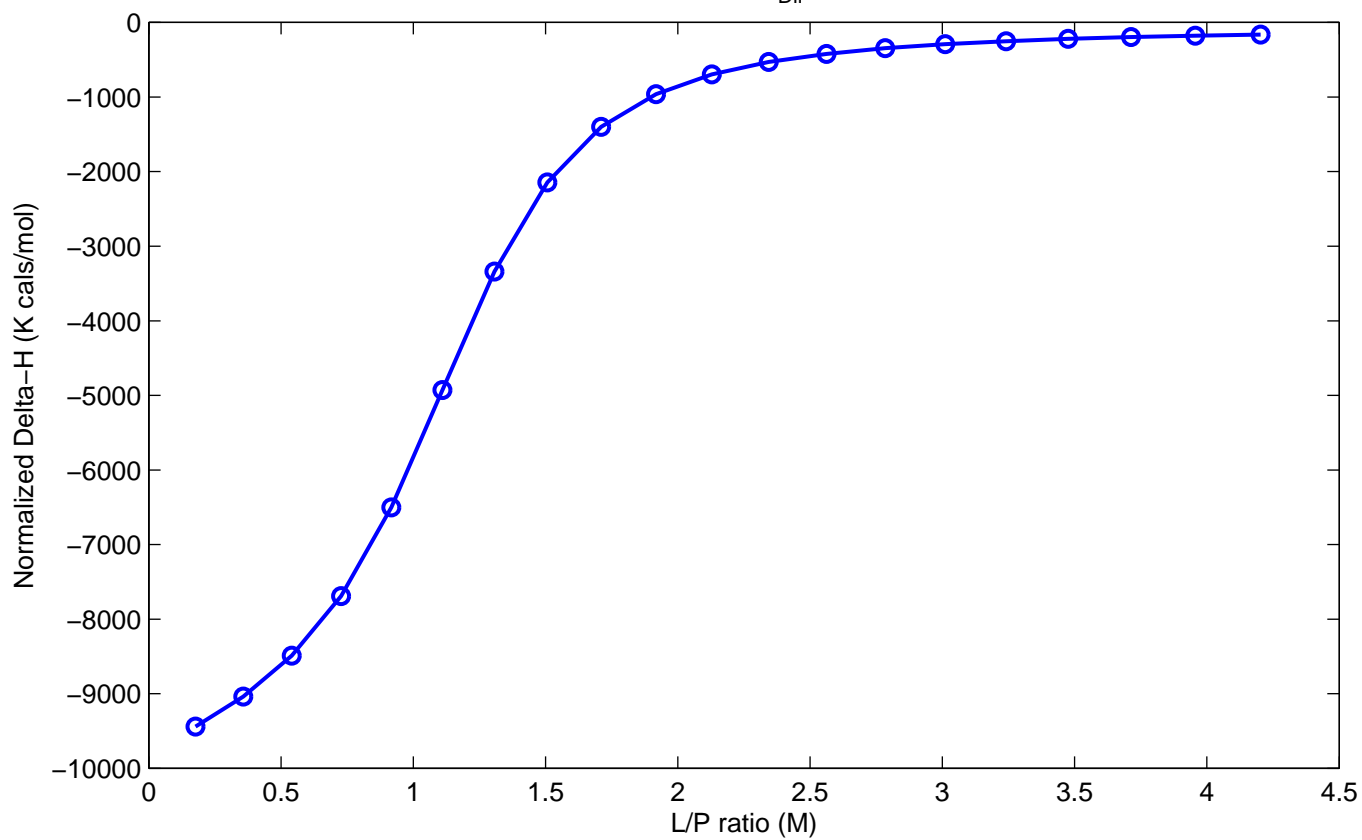

Supplement: Multimedia component 2 [file mmc2.zip › SI_Figure_S1/100_Pts/Processed_data.pdf]

Two state

$\tau_L: 3 \text{ (s)}$   $\tau_{\Delta H}: 3 \text{ (s)}$   $\tau_{\Delta H_{Dil}}: 3 \text{ (s)}$

$K_{eq}: 1.0e+06$   $k_{on}: 1.0e+06$   $k_{off}: 1.0e+00$

$\Delta H: -1.0e+04$   $\Delta H_{Dil}: -1.0e+02$

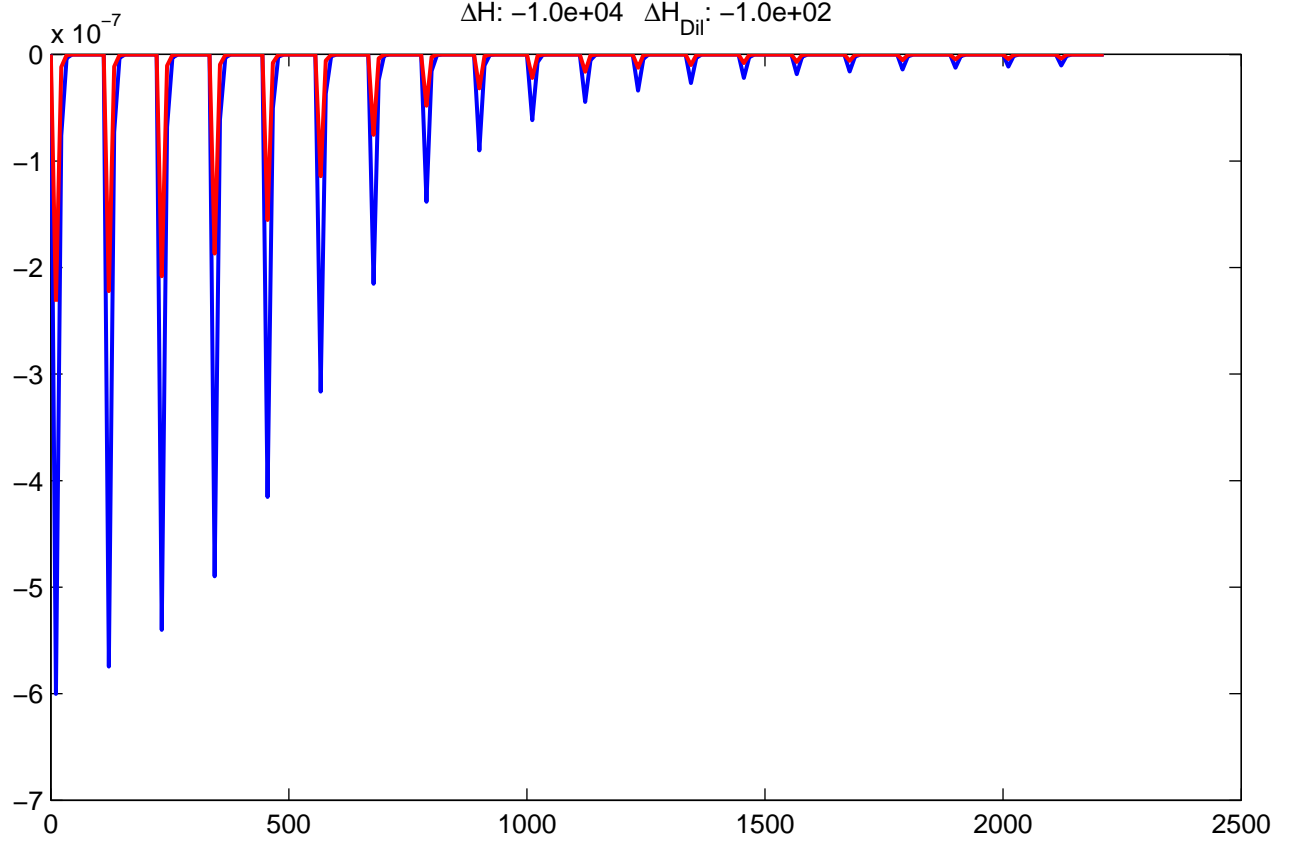

Supplement: Multimedia component 2 [file mmc2.zip › SI_Figure_S1/10_Pts/Chromatogram.pdf]

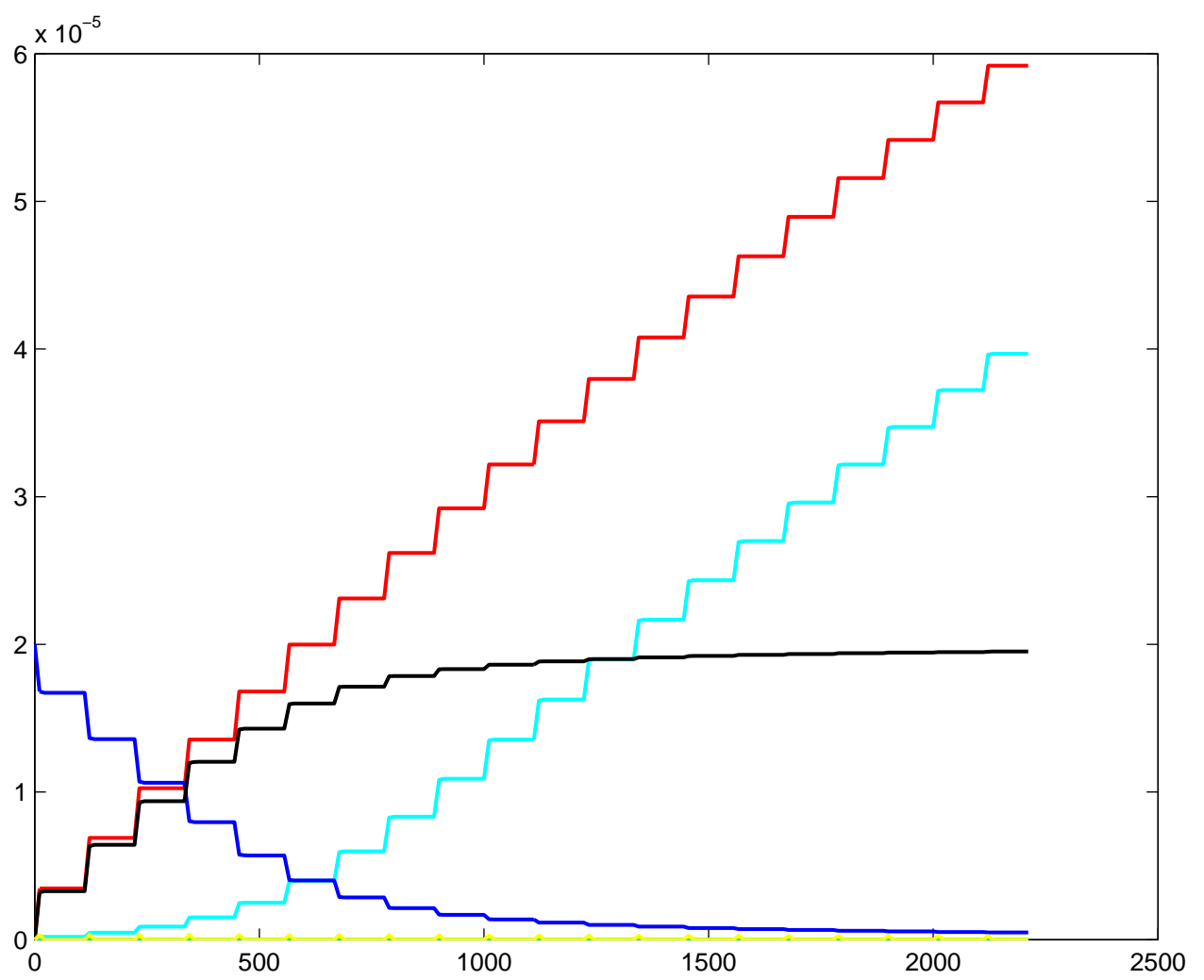

Supplement: Multimedia component 2 [file mmc2.zip › SI_Figure_S1/10_Pts/Kinetics(P,PL,L).pdf]

Two state

$\tau_L$ : 3 (s)  $\tau_{\Delta H}$ : 3 (s)  $\tau_{\Delta H_{Dil}}$ : 3 (s)

$K_{eq}$ : 1.0e+06  $k_{on}$ : 1.0e+06  $k_{off}$ : 1.0e+00

$\Delta H$ : -1.0e+04  $\Delta H_{Dil}$ : -1.0e+02

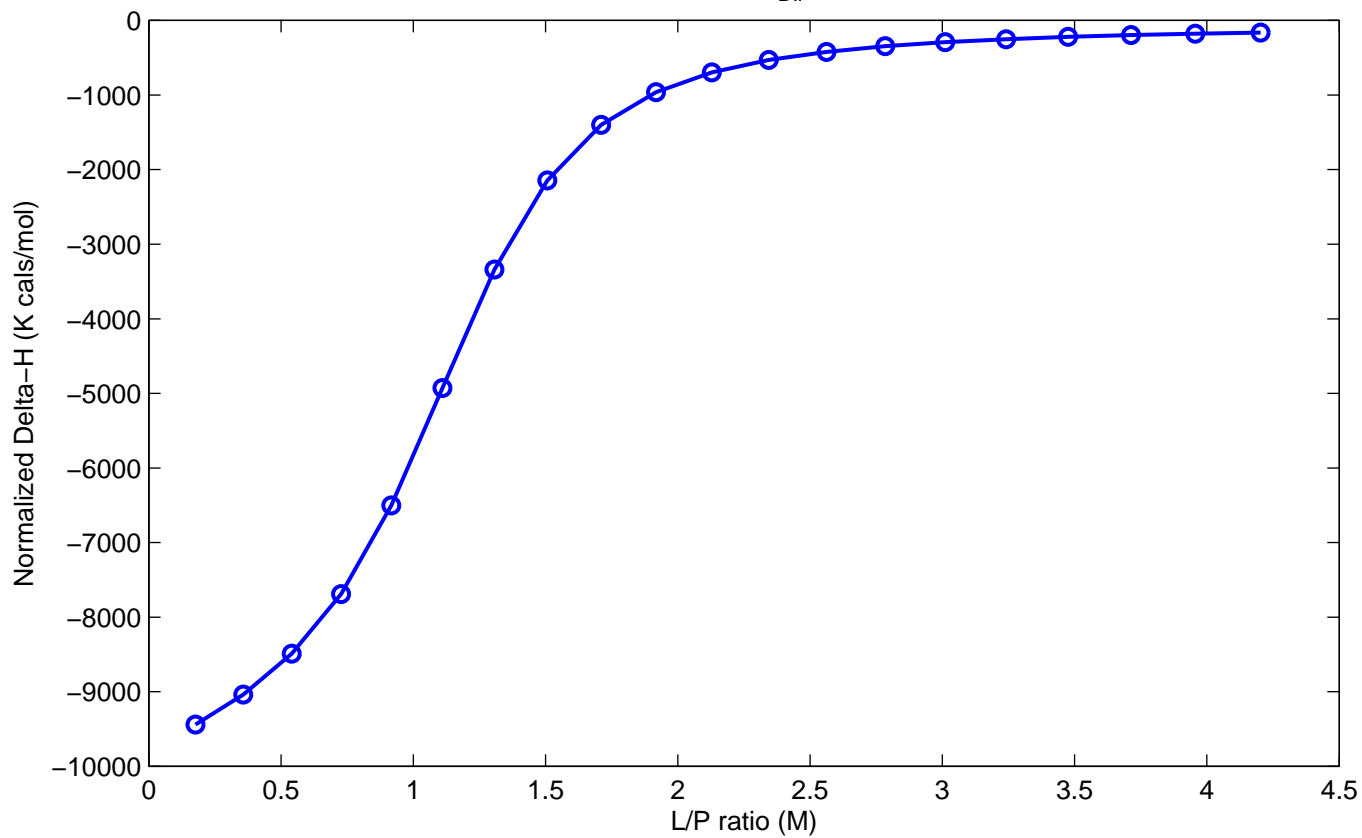

Supplement: Multimedia component 2 [file mmc2.zip › SI_Figure_S1/10_Pts/Processed_data.pdf]

Two state

$\tau_L: 3 \text{ (s)}$   $\tau_{\Delta H}: 3 \text{ (s)}$   $\tau_{\Delta H_{Dil}}: 3 \text{ (s)}$

$K_{eq}: 1.0e+06$   $k_{on}: 1.0e+06$   $k_{off}: 1.0e+00$

$\Delta H: -1.0e+04$   $\Delta H_{Dil}: -1.0e+02$

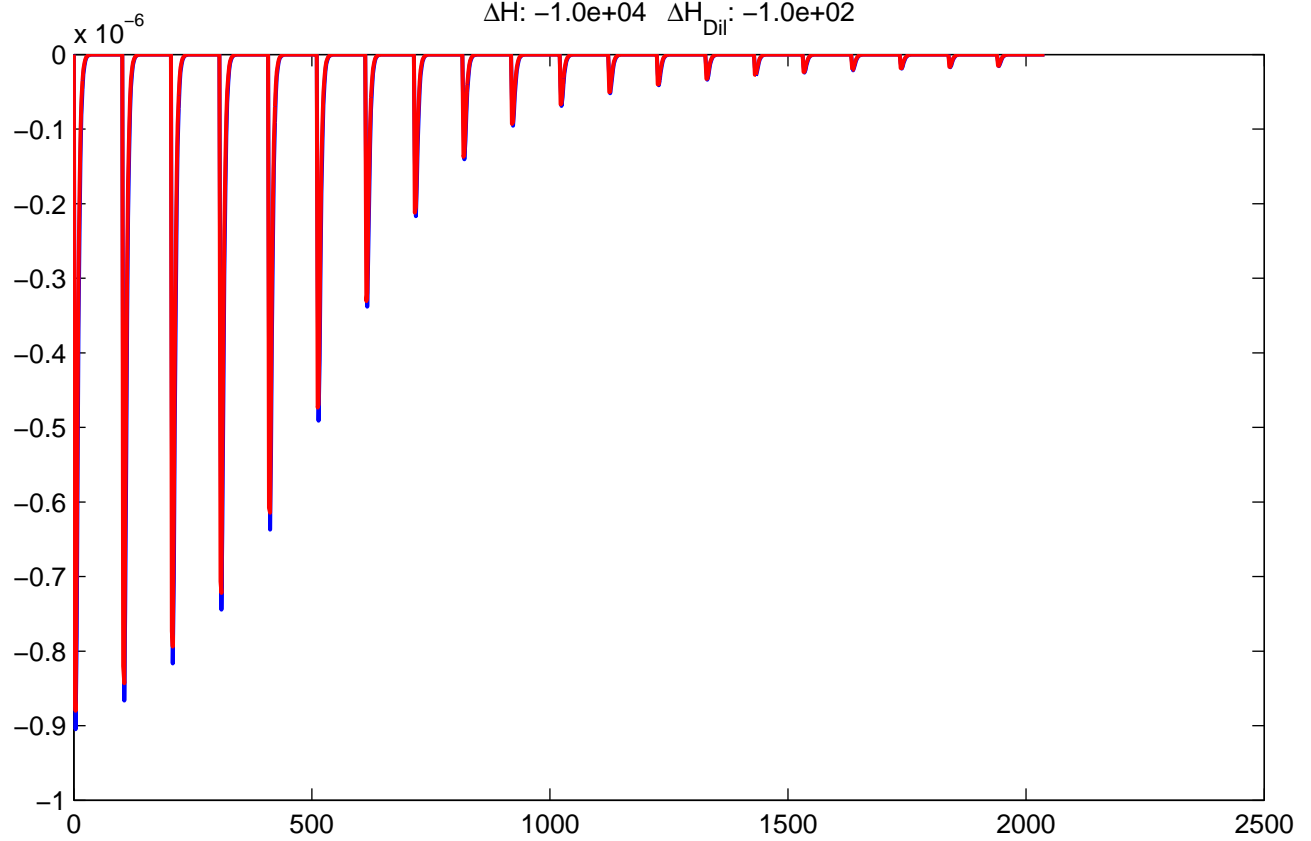

Supplement: Multimedia component 2 [file mmc2.zip › SI_Figure_S1/50_Pts/Chromatogram.pdf]

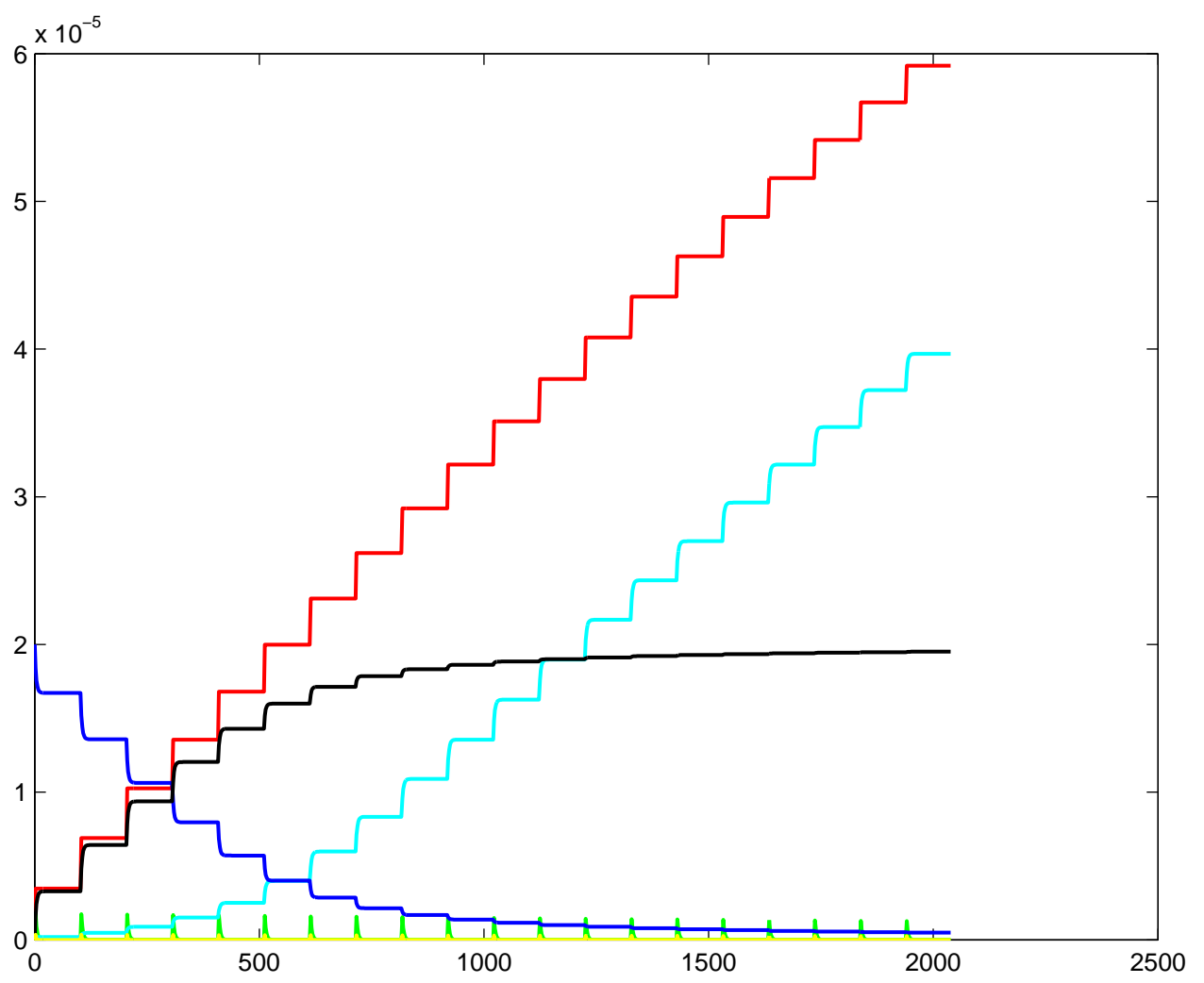

Supplement: Multimedia component 2 [file mmc2.zip › SI_Figure_S1/50_Pts/Kinetics(P,PL,L).pdf]
