# Supplementary material for: Isothermal titration calorimetry and surface plasmon resonance analysis using the dynamic approach
Source: Biochem Biophys Rep. 2019 Dec 17;21:100712. doi: 10.1016/j.bbrep.2019.100712 (PMC6926116; doi:10.1016/j.bbrep.2019.100712)

Two state

τ_L: 3 (s) $\tau_{\Delta H}$: 3 (s) $\tau_{\Delta H_{Dil}}$: 3 (s)

K_{eq}: 1.0e+06 k_{on}: 1.0e+06 k_{off}: 1.0e+00

ΔH: −1.0e+04 ΔH_{Dil}: −1.0e+02

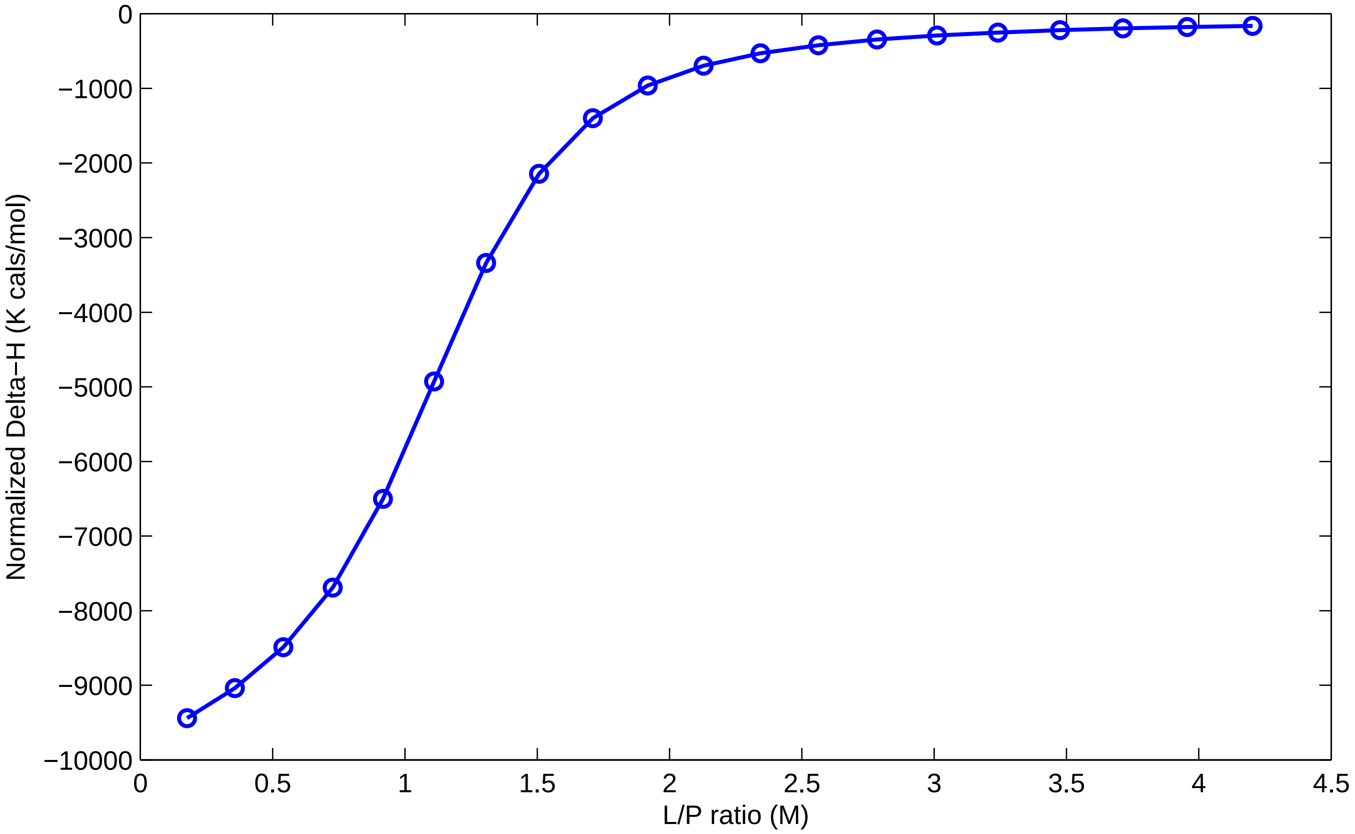

Supplement: Multimedia component 2 [file mmc2.zip › SI_Figure_S1/50_Pts/Processed_data.pdf]
