# Supplementary material for: Isothermal titration calorimetry and surface plasmon resonance analysis using the dynamic approach
Source: Biochem Biophys Rep. 2019 Dec 17;21:100712. doi: 10.1016/j.bbrep.2019.100712 (PMC6926116; doi:10.1016/j.bbrep.2019.100712)
Supplement: Multimedia component 2 [file mmc2.zip › SI_Figure_S2/Prot_agg/Abstracted_method/Chromatogram.pdf]

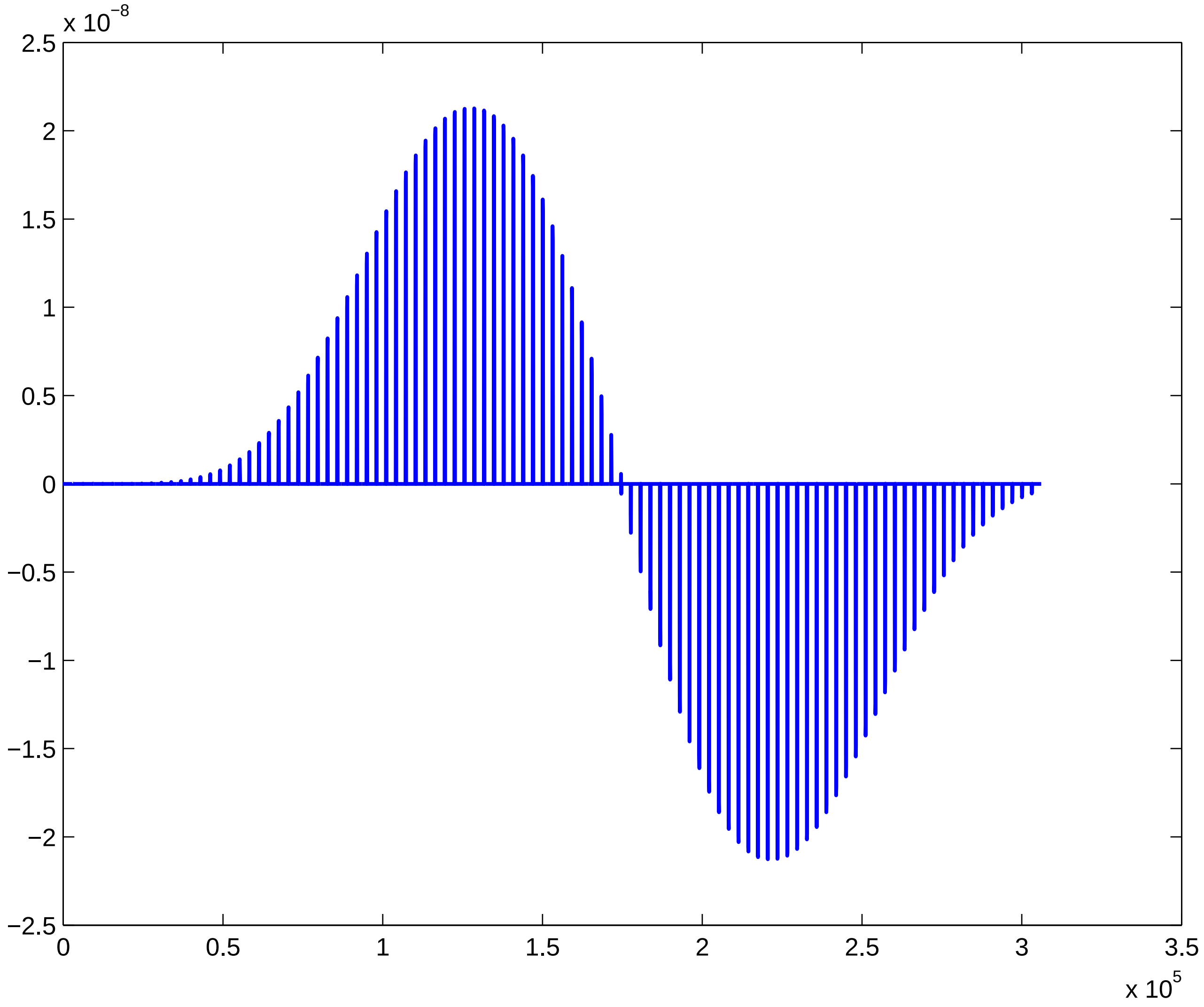
x 10^−8
2.5
2
1.5
1
0.5
0
−0.5
−1
−1.5
−2
−2.5
0
0.5
1
1.5
2
2.5
3
3.5
x 10^5
