# Supplementary material for: Isothermal titration calorimetry and surface plasmon resonance analysis using the dynamic approach
Source: Biochem Biophys Rep. 2019 Dec 17;21:100712. doi: 10.1016/j.bbrep.2019.100712 (PMC6926116; doi:10.1016/j.bbrep.2019.100712)
Supplement: Multimedia component 2 [file mmc2.zip › SI_Figure_S2/Prot_agg/Abstracted_method/Kinetics(P,PL,L).pdf]

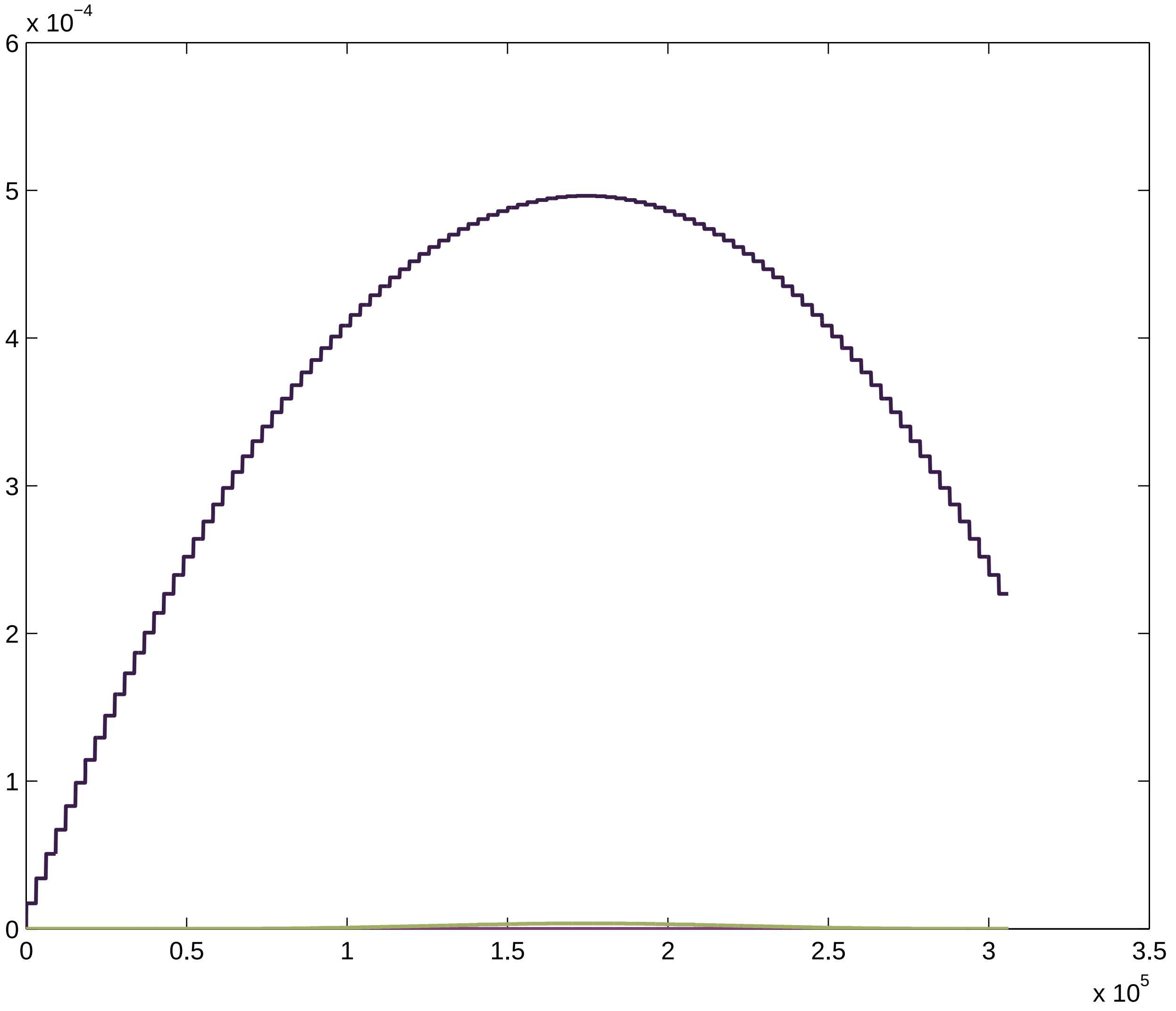

x 10^−4
6
5
4
3
2
1
0
0
0.5
1
1.5
2
2.5
3
3.5
x 10^5
