# Supplementary material for: Isothermal titration calorimetry and surface plasmon resonance analysis using the dynamic approach
Source: Biochem Biophys Rep. 2019 Dec 17;21:100712. doi: 10.1016/j.bbrep.2019.100712 (PMC6926116; doi:10.1016/j.bbrep.2019.100712)

Two state

τ_L: 1 (s) $\tau_{\Delta H}$: 1 (s)

K_{eq}: 1.0e+52 k_{on}: 1.0e+52 k_{off}: 1.0e+00

ΔH P−intra: 1.0e+04

ΔH_{Dil}: 0.0e+00

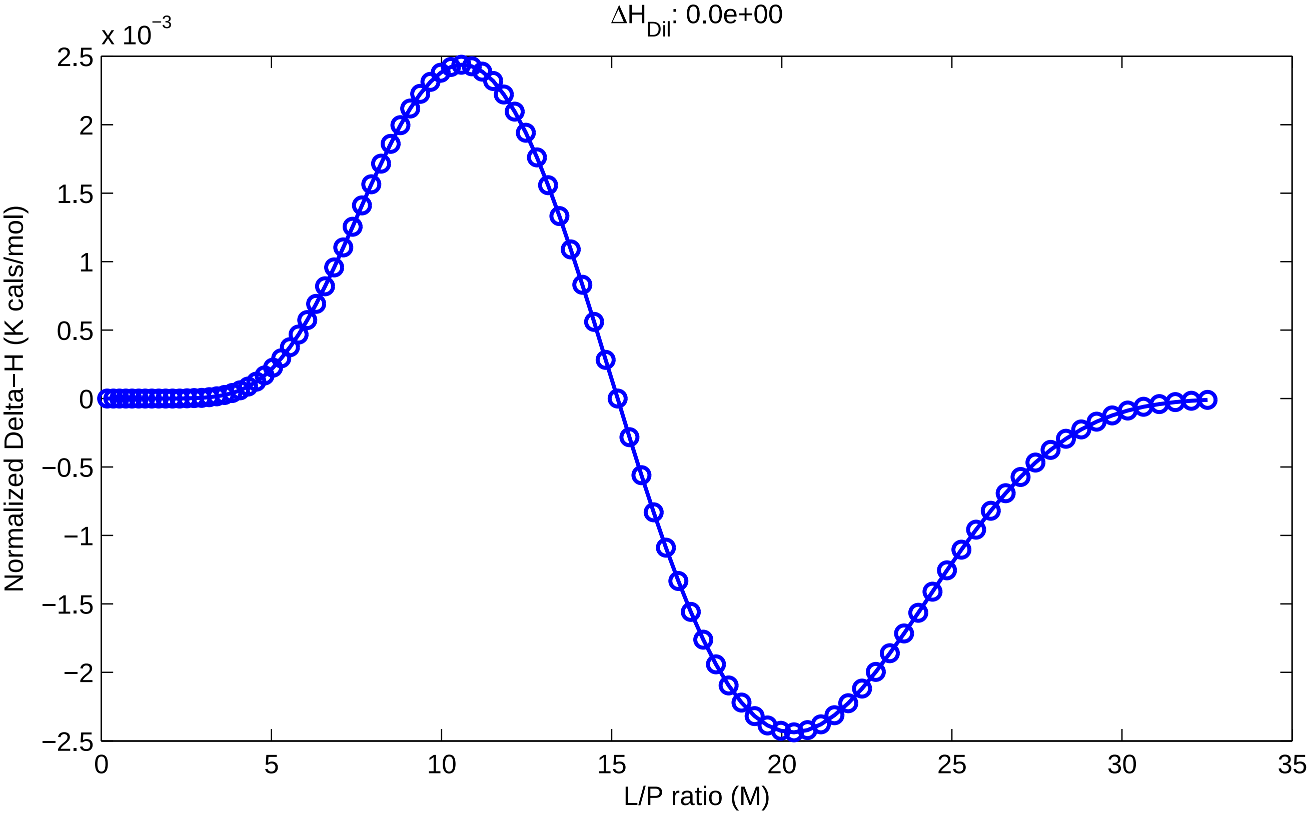

Supplement: Multimedia component 2 [file mmc2.zip › SI_Figure_S2/Prot_agg/Explicit_method/Explicit_16Mer/Processed_data.pdf]
