# Supplementary material for: Isothermal titration calorimetry and surface plasmon resonance analysis using the dynamic approach
Source: Biochem Biophys Rep. 2019 Dec 17;21:100712. doi: 10.1016/j.bbrep.2019.100712 (PMC6926116; doi:10.1016/j.bbrep.2019.100712)
Supplement: Multimedia component 2 [file mmc2.zip › SI_Figure_S2/Prot_agg/Explicit_method/Explicit_2Mer/Processed_data.pdf]

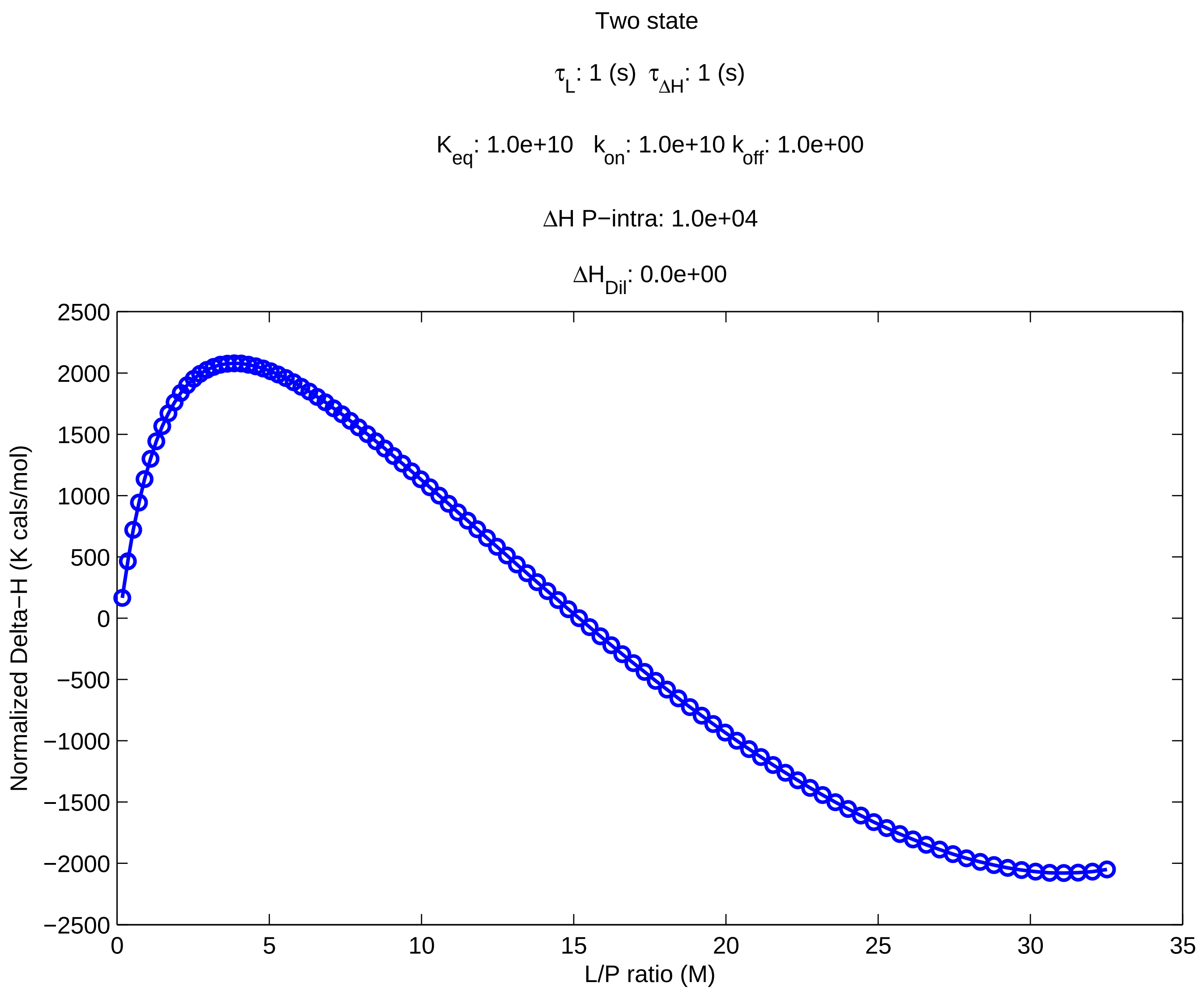
Two state
τL: 1 (s) τΔH: 1 (s)
Keq: 1.0e+10 kon: 1.0e+10 koff: 1.0e+00
ΔH P−intra: 1.0e+04
ΔHDil: 0.0e+00
Normalized Delta−H (K cals/mol)
L/P ratio (M)
