# Supplementary material for: Isothermal titration calorimetry and surface plasmon resonance analysis using the dynamic approach
Source: Biochem Biophys Rep. 2019 Dec 17;21:100712. doi: 10.1016/j.bbrep.2019.100712 (PMC6926116; doi:10.1016/j.bbrep.2019.100712)
Supplement: Multimedia component 2 [file mmc2.zip › SI_Figure_S2/Prot_agg/Explicit_method/Explicit_4Mer/Chromatogram.pdf]

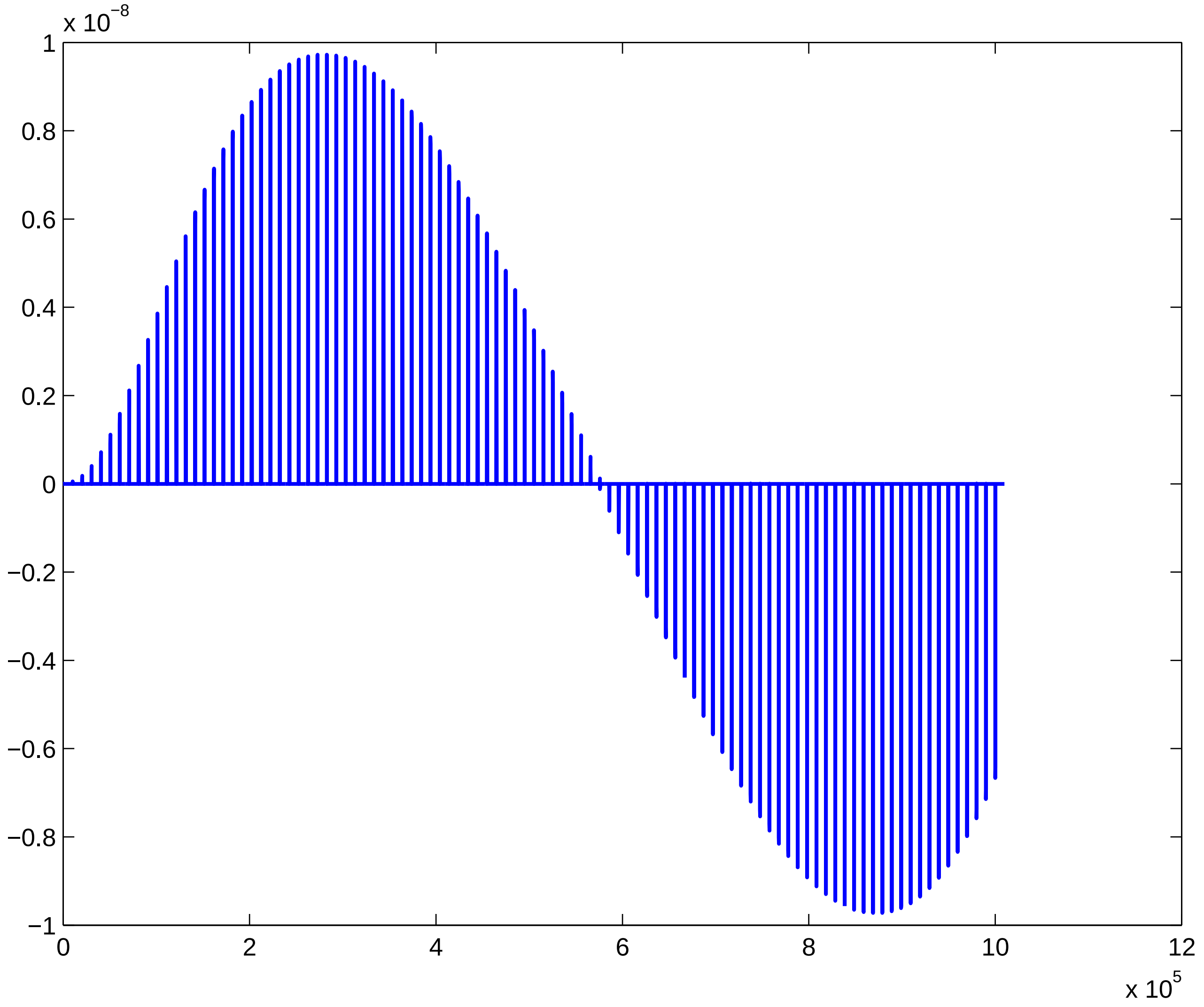

x 10^−8
1
0.8
0.6
0.4
0.2
0
−0.2
−0.4
−0.6
−0.8
−1
0
2
4
6
8
10
12
x 10^5
